# Unraveling the brain dynamics of Depersonalization-Derealization Disorder: a dynamic functional network connectivity analysis

**DOI:** 10.1186/s12888-024-06096-1

**Published:** 2024-10-14

**Authors:** Sisi Zheng, Francis Xiatian Zhang, Hubert P. H. Shum, Haozheng Zhang, Nan Song, Mingkang Song, Hongxiao Jia

**Affiliations:** 1 Beijing Key Laboratory of Mental Disorders, National Clinical Research Center for Mental Disorders & National Center for Mental Disorders, Beijing Anding Hospital, Capital Medical University, Beijing, 100088 China; 2 Advanced Innovation Center for Human Brain Protection, Capital Medical University, Beijing, 100069 China; 3 Department of Computer Science, Durham University, Durham, DH1 3LE UK

**Keywords:** Functional Network Connectivity, FMRI, Depersonalization-Derealization Disorder

## Abstract

**Background:**

Depersonalization-Derealization Disorder (DPD), a prevalent psychiatric disorder, fundamentally disrupts self-consciousness and could significantly impact the quality of life of those affected. While existing research has provided foundational insights for this disorder, the limited exploration of brain dynamics in DPD hinders a deeper understanding of its mechanisms. It restricts the advancement of diagnosis and treatment strategies. To address this, our study aimed to explore the brain dynamics of DPD.

**Methods:**

In our study, we recruited 84 right-handed DPD patients and 67 healthy controls (HCs), assessing them using the Cambridge Depersonalization Scale and a subliminal self-face recognition task. We also conducted a Transcranial Direct Current Stimulation (tDCS) intervention to understand its effect on brain dynamics, evidenced by Functional Magnetic Resonance Imaging (fMRI) scans. Our data preprocessing and analysis employed techniques such as Independent Component Analysis (ICA) and Dynamic Functional Network Connectivity (dFNC) to establish a comprehensive disease atlas for DPD. We compared the brain's dynamic states between DPDs and HCs using ANACOVA tests, assessed correlations with patient experiences and symptomatology through Spearman correlation analysis, and examined the tDCS effect via paired t-tests.

**Results:**

We identified distinct brain networks corresponding to the Frontoparietal Network (FPN), the Sensorimotor Network (SMN), and the Default Mode Network (DMN) in DPD using group Independent Component Analysis (ICA). Additionally, we discovered four distinct dFNC states, with State-1 displaying significant differences between DPD and HC groups (*F* = 4.10, *P* = 0.045). Correlation analysis revealed negative associations between the dwell time of State-2 and various clinical assessment factors. Post-tDCS analysis showed a significant change in the mean dwell time for State-2 in responders (*t*-statistic = 4.506, *P* = 0.046), consistent with previous clinical assessments.

**Conclusions:**

Our study suggests the brain dynamics of DPD could be a potential biomarker for diagnosis and symptom analysis, which potentially leads to more personalized and effective treatment strategies for DPD patients.

**Trial registrations:**

The trial was registered at the Chinese Clinical Trial Registry on 03/01/2021 (Registration number: ChiCTR2100041741, https://www.chictr.org.cn/showproj.html?proj=66731) before the trial.

**Supplementary Information:**

The online version contains supplementary material available at 10.1186/s12888-024-06096-1.

## Backgrounds

Depersonalization-derealization disorder (DPD), a prevalent psychiatric disorder, fundamentally disrupts self-consciousness and could significantly impact the quality of life of those affected It manifests as a detachment from one's environment and personal self, which affects approximately 1% of the wider population [1, 2]. This prevalence rises to 5%-20% among outpatients and further increases to 17.5–41.9% in inpatient settings [1]. DPD patients frequently experience a disturbing sensation of being in a dreamlike state or questioning their existence [3]. The core of the symptoms lies in a disruption in self-referential processing [4], a fundamental aspect of consciousness [4]. Illustrative experiments, such as the rubber hand illusion study [5], reveal that individuals with pronounced DPD tendencies demonstrate altered visual-tactile integration. Additionally, diminished experiences of the full-body illusion in self-association contexts further underscore this anomaly [6].

While existing research has provided foundational insights into this disorder [7], the limited exploration of brain dynamics in DPD hinders a deeper understanding of its mechanisms and restricts the advancement of diagnostic and treatment strategies. Dynamic Functional Network Connectivity (dFNC) has recently emerged as a popular approach to exploring brain dynamics by analyzing the evolving relationships between functional regions [8–10], which may be also a suitable method to explore the brain dynamics in DPD. Specifically, brain regions with similar functional specializations exhibit Functional Connectivity (FC) through correlated blood oxygen level-dependent signals during rest [11], indicating a strong link between them. FC could be a biomarker for diagnoses [12–14] or efficacy of treatment [15]. These functionally similar brain regions constitute the brain network. Large-scale dFNC offers a more direct, context-sensitive, and dynamic view at a higher network level [16]. Moreover, the brain regions altered in the DPD found in the previous research were parts of the Default Mode Network (DMN), the Sensorimotor Network (SMN), or the Frontoparietal Network (FPN). The right Anterior Cingulate Cortex (ACC) [17], bilateral Medial Prefrontal Cortex (mPFC) [17], and temporoparietal junction [18–21] are part of DMN; the insula [22] is part of SMN; the Dorsolateral Prefrontal Cortex (dlPFC) [23] is part of the FPN [24, 25]. The SMN [26], FPN [27, 28], and DMN [29, 30] also emerge as central players in consciousness studies. These networks are crucial for understanding the workings of the consciousness. The SMN facilitates consciousness within the brain's overarching functional architecture [31], while DMN-FPN connectivity is vital for introspective cognition [32]. Despite this, there is still a notable gap in the study of dFNC in DPD patients. Building on this existing evidence, our paper primarily focuses on exploring brain dynamics within and between the SMN, FPN, and DMN.

**Fig. 1:**
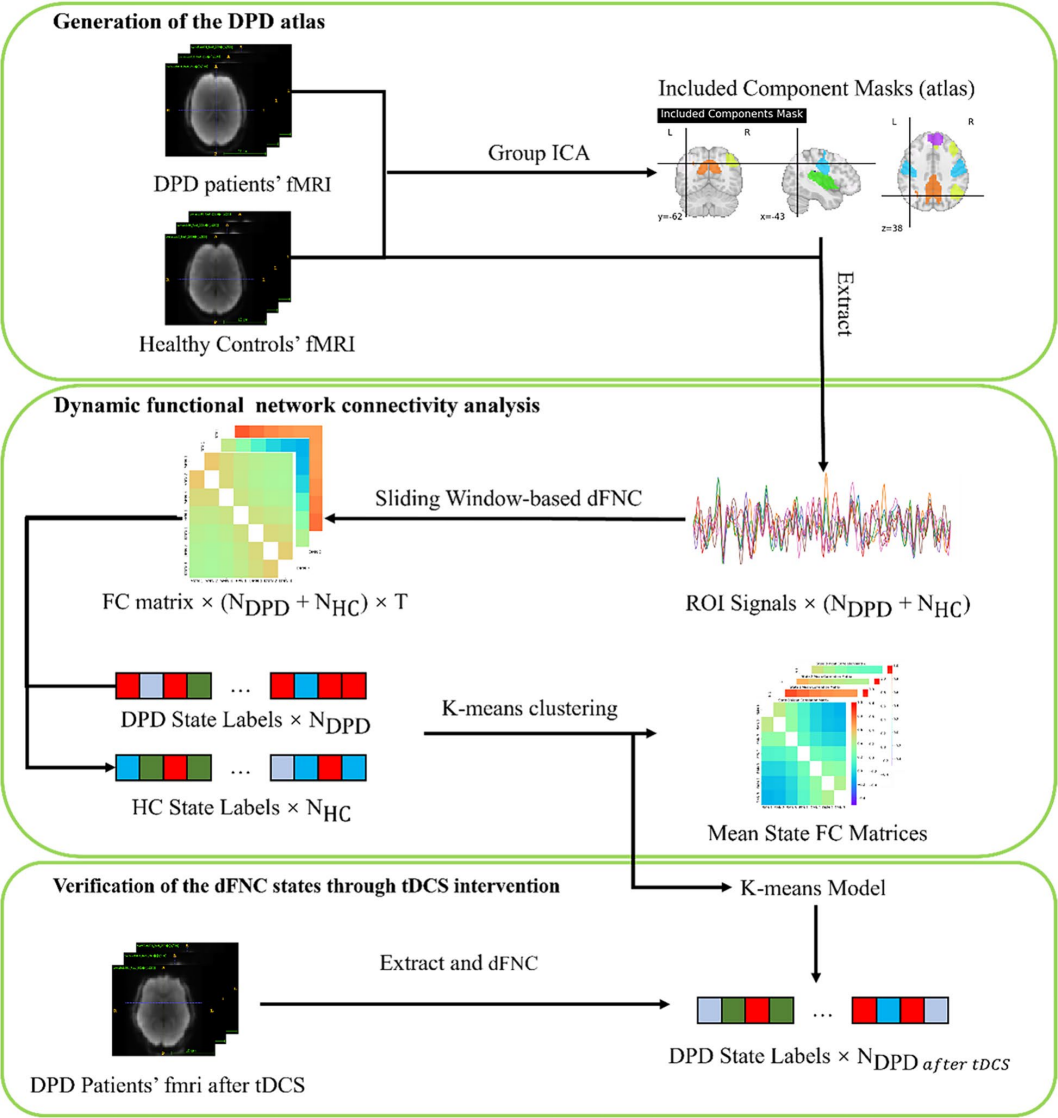
Methods (Data Analysis). ${N}_{DPD}$ denotes the number of DPD patients, ${N}_{HC}$ denotes the number of HCs, T denotes the time windows number extracted for each single scan, and ${N}_{DPD after tDCS}$ denotes the number of DPD patients after tDCS treatment. First, we adapt the group ICA to generate a DPD atlas from the fMRI of the DPD group and the HC group, which identifies intrinsic connectivity network. Second, we employ our atlas and K-means cluster method to get the brain dynamics, which support our detailed dFNC analysis. Third, we incorporated observations of patients' neural dynamics following Transcranial Direct Current Stimulation (tDCS) intervention, to verify our dFNC findings for DPD

To address the limited exploration of brain dynamics in DPD, our study conducts a comprehensive dFNC analysis focusing on diagnosis and symptoms. We compared the dFNC in DPD patients and healthy controls (HCs). We then performed the correlation analyses between the dFNC states with the clinical assessments among DPD patients, containing the symptoms measured by the CDS and the level of self-consciousness assessed by a subliminal self-face recognition task. To verify our findings of brain dynamics in DPD, we incorporated observations of patients' brain dynamics following Transcranial Direct Current Stimulation (tDCS) intervention, a neuroregulation technique. tDCS has already shown potential benefits for individuals with various mental conditions, such as stress-related mental health [33] Thus, we checked whether dFNC states differed before and after tDCS interventions in responsive patients. Figure 1 illustrates the workflow of our data analysis. Advanced techniques, including Independent Component Analysis (ICA) and dFNC, were incorporated to delve into brain dynamics. Through the employment of the sliding window, clustering, and correlation techniques, dynamic state labels were determined for HC participants, DPD patients, and DPD patients post-tDCS intervention. Leveraging these state labels, which could be viewed as brain dynamics, evaluations were conducted to understand the influence of brain dynamics on the personal experiences of DPD patients and their change post-tDCS.

## Methods

### Participants

We recruited 84 right-handed patients diagnosed with DPD from the Outpatient Department of Beijing Anding Hospital, Capital Medical University. All examinations were conducted following the principles of the Declaration of Helsinki. The Ethics Committee of Beijing Anding Hospital, Capital Medical University, China approved the present study. Some of the data (DPD samples) presented in this paper have been previously published in our study [34].

DPD patients were diagnosed according to ICD-10 and screened through the Chinese version of the Dissociative Disorders Interview Schedule (DDIS, https://www.rossinst.com/ddis, accessed on 5 September 2018) [35] and the mini-international neuropsychiatric interview (M.I.N.I.) [36]. Patients with DPD were included if they were (a) 15–45 years old, (b) right-handed, and (c) with a score of Cambridge Depersonalization Scale (CDS) [37] ≥ 70. They were excluded if they had (a) transient experiences of Depersonalization and/or Derealization due to trauma, fatigue, or substance use; (b) a history of neurological disorders or family history of hereditary neurological disorders; (c) history of substance addiction or brain trauma; (d) gross morphological anomalies, as evidenced by brain MRI; and (e) any electronic or metal implants.

67 HCs matched with age, sex and education were recruited by the solicitation, screened through M.I.N.I, and interviewed for the 2-item CDS [3] to determine the absence of previous DPD symptoms or psychiatric diseases. None of the HCs had reported a history of acute or chronic illness.

### Clinical assessment and self-referential processing task

The CDS was developed by Sierra and Berrios specifically for DPD and consists of 29 self-reported items [37]. Each item encompasses both duration and frequency sub-items. In clinical studies, a score of 70 is typically used as a threshold, with scores above 70 exhibiting a sensitivity of 75.5% and a specificity of 87.2%. While previous factor analyses have shown that the CDS is multidimensional, there is no large-sample unified factor structure, and there is a lack of factor analysis in the Chinese population.

In this study, we utilized three versions of the scale: the original 4-factor version from the scale's development team [38], a subsequently derived 5-factor version that exhibited good results [39], and a more simplistic 2-factor version [40]. The 4-factor version comprises Anomalous Body Experience, Emotional Numbing, Anomalous Subjective Recall, and Alienation from Surroundings. The 5-factor version includes Numbing, Unreality of Self, Perceptual Alterations, Unreality of Surroundings, and Temporal Disintegration. The 2-factor version consists of factors representing a sense of unreality and detachment, and emotional and physical numbing.

### Self-referential processing task

To reflect the level of self-awareness of the subjects, this study employed the time taken by the test group in a subliminal self-face recognition task as an indicator of the level of self-awareness in DPD patients. The task utilized a continuous flash suppression (CFS) experimental paradigm. CFS is an experimental technique involving dynamic noise images presented to the dominant eye and a face image with increasing contrast to the non-dominant eye. This induces interocular suppression, making the face initially imperceptible. As time progresses and face contrast increases, it breaks through suppression into consciousness. The time taken defines subliminal processing. Thus, we used this task as an indicator of the level of self-awareness in DPD patients.

**Fig. 2:**
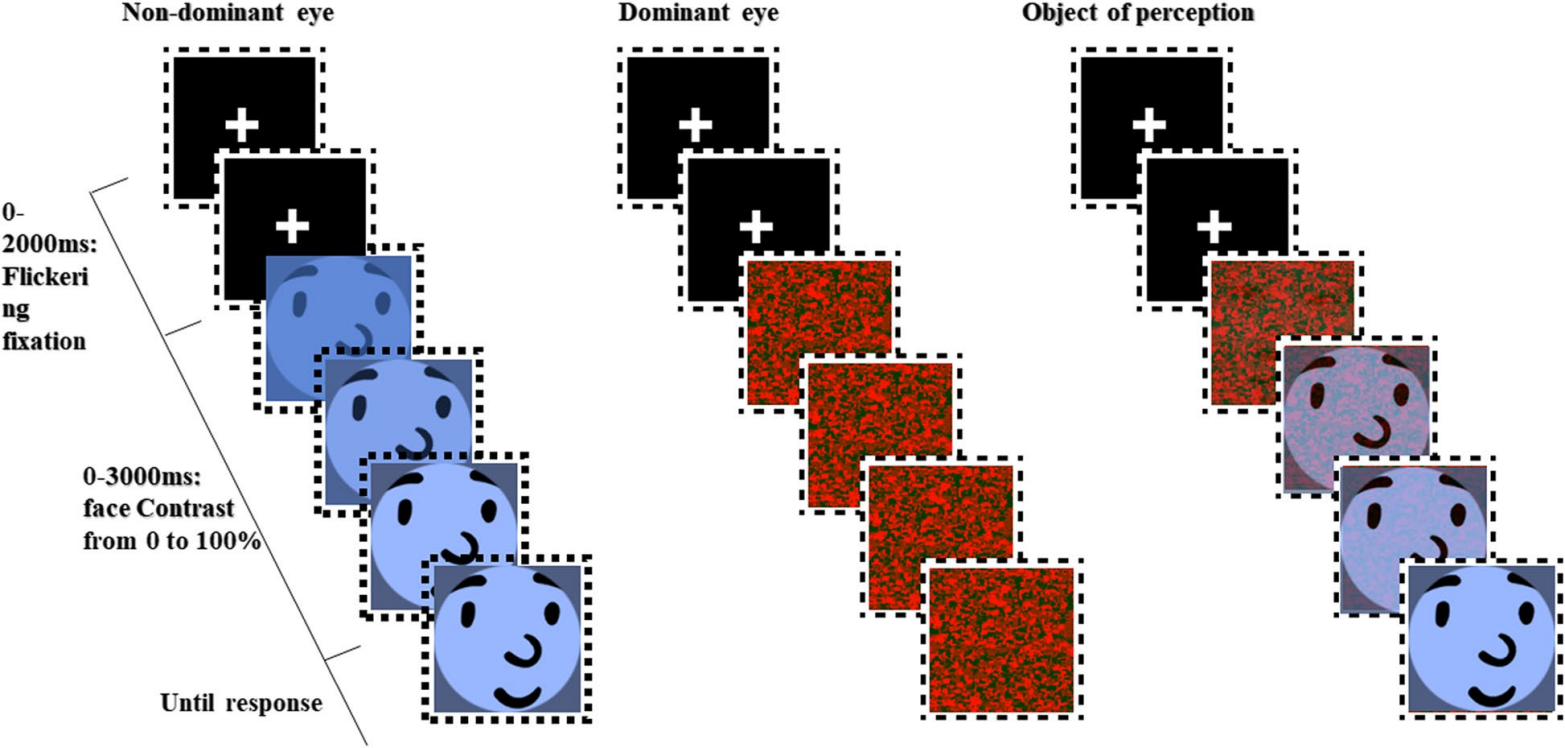
Illustration of a trial in the experimental paradigm. In the experimental condition, the non-dominant eye was exposed to the participant’s own face or scrambled pictures, transitioning from 0 to 100% contrast over 1000 ms and then remaining at 100% for 9000 ms, while the dominant eye was exposed to noise images at 100% contrast. In the control condition, both eyes were exposed to images that varied from 0 to 100% contrast, maintaining 100% for 4000 ms

The illustration of the experiment is shown in Fig. 2. All participants were tested in a soundproof laboratory and completed the experiment after understanding the requirements and practicing until confident. During the experiment, participants’ heads were fixed with a chin rest 60 cm away from a computer screen.

The experiment consisted of two blocks: an experimental condition and a control condition. In the experimental block, participants wore red-blue anaglyph glasses, with noise images visible to the dominant eye through the red lens and face images to the other eye through the blue lens. This setup created interocular suppression, allowing participants to see continuous noise images while the face image remained suppressed until breaking into awareness. In the control condition, without anaglyph glasses, noise and face images were presented to both eyes simultaneously, eliminating binocular suppression.

Each block included 55 trials: 15 self-face pictures, 15 famous faces, 15 stranger faces, and 10 scrambled faces. Scrambled pictures were shown to the non-dominant eye, and a response within 10 seconds marked an error. After three false alarms, the trial restarted. Trials began with a white fixation point for 2000 ms to attract attention. In the experimental condition, the dominant eye saw 100% contrast noise images, while the non-dominant eye saw face or scrambled pictures, transitioning from 0 to 100% contrast over 1000 ms and remaining at 100% for 9000 ms. In the control condition, both eyes saw images varying from 0 to 100% contrast, maintaining 100% for 4000 ms.

Participants pressed the space bar as quickly as possible upon recognizing the face image. Images remained on the screen until a response or 10 seconds elapsed. Details of the task can be found in a previously published paper [4]. We used the mean response time of self-faces in the experimental condition to represent the level of self-consciousness of patients.

### tDCS intervention

The experiment utilized the DC-STIMULATOR direct current microelectrode stimulator manufactured by Germany's NeuroConn company in conjunction with conductive rubber electrodes measuring 5 × 7 cm^2^. Sponge covers were soaked in a dilute NaCl saline solution to enhance conductivity and then placed over the conductive rubber. A gradient method was employed to prevent the sudden onset and offset of current at the start and end of stimulation, which could cause pronounced stinging or discomfort for the participant. The current gradually peaked over 15 seconds, with stimulation parameters set at 2 mA and lasting 20 min. Electrode placement on the skull was determined based on the 10–20 EEG system. The stimulation site for the anode targeted the dlPFC at the F3 location, which is supported by imaging evidence [41]. In the anodal stimulation mode, the anode electrode was placed on F3, while the cathode electrode was positioned on F4. The inclusion criteria for study participants remained consistent with previous standards. The Functional Magnetic Resonance Imaging (fMRI) was taken before the tDCS intervention, and a second fMRI was captured within 30 min after a single intervention to explore the mechanism of tDCS intervention. Patients with a reduction of more than 25% in their CDS score after 10 sessions of tDCS therapy were considered responders. A reduction of 25% in the CDS score after ten tDCS therapy sessions was considered adequate.

### Data acquisition and preprocessing

#### Image acquisition

Resting-state fMRI data were acquired with a 3.0 Tesla MRI scanner (Prisma 3.0; Siemens, Germany) in the Beijing Anding Hospital, Capital Medical University, China. fMRI data were acquired with a single-shot, gradient-recalled echo-planar imaging sequence with the following parameters: repetition time = 2000 ms, echo time = 30 ms, flip angle = 90°, matrix = 64 × 64, field of view = 200 mm × 200 mm, slice thickness = 3.5 mm, gap = 1 mm, 33 axial sections, and 240 volumes.

High-resolution brain structural images were acquired with a T1-weighted three-dimensional (3D) multi-echo magnetization-prepared rapid gradient-echo (MPRAGE) sequence (echo time: 3.39 ms, repetition time: 2530 ms, slice thickness 1.3 mm, voxel size: 1.3 × 1 × 1 mm3, field of view (FOV): 256 × 256 mm2 and volume number: 176).

Participants were required to undergo a 30-min rest period before scanning. They were explicitly instructed to remain still and awake throughout the scanning session. Foam head holders were used to immobilize participants during the scanning process.

#### fMRI preprocessing

All image preprocessing was completed by DPABI [42] and SPM 12 [43]. The resting-state functional MRI (fMRI) data were preprocessed with the following steps: removal of the first ten volumes, correction for slice timing, realignment to the first volume, and spatial normalization within the native space. Subsequently, echo-planar imaging volumes were co-registered to the corresponding T1-weighted MRI images, normalized to MNI space using the normalization parameters derived from the T1-weighted MRI, and smoothed with a 4-mm full-width at half-maximum Gaussian kernel.

#### The generation of the DPD atlas

To generate the DPD atlas, we leverage the CanICA function from the Nilearn library to perform Independent Component Analysis (ICA) on the collected neuroimaging data [44]. This process involves defining 30 independent components and deploying a 'whole-brain-template' mask strategy. Notably, the number of components was chosen as suggested by the former research that higher model orders in ICA can capture finer-grained sub-networks and enhance sensitivity to subtle connectivity changes [45]. We did not opt for a higher number of components in our ICA analysis because increasing the model order beyond 30 can lead to overfitting and a reduction in the reproducibility of the identified components. Overfitting occurs when the model becomes excessively complex, capturing noise instead of meaningful signals, which distorts the true functional connectivity patterns. Additionally, higher model orders may yield components that are less interpretable and more difficult to relate to known neuroanatomical structures or functional networks.

To identify the brain regions involved in each component, we used the RegionExtractor tool from Nilearn's regions module. The resulting data are preserved as NIfTI images, which are subsequently analyzed. Functional connectivity between components was calculated as the mean of pairwise functional connectivity between the regions identified by the RegionExtractor. This approach ensures that the connectivity measures reflect the interactions between the specific regions within each component. Following the ICA decomposition, we utilize the Yeo 2011 7-network atlas for network extraction [46], selecting SMN, FPN and DMN for further scrutiny. We then calculate the similarity between the derived ICA components and the selected networks. This process involves applying a mask to the components associated with each network and computing the percentage overlap. We identify the components with the most significant similarity for each network, given that they exceed a specified threshold of 50%.

### dFNC analysis

#### dFNC extraction

With our generated DPD atlas, we employ the sliding window technique to generate dynamic correlation matrices that encapsulate the FC patterns in discrete time windows. We first normalize the ROI signals using a convolution with sigma = 1 for smoothing and then define the window size as 20 and step size as 3. This choice is informed by previous work indicating that a window length between 30 and 60 seconds is optimal for estimating dFNC [8]. The generated matrices rely on correlation analysis, a commonly employed method in dFNC studies [47]. After this step, we normalize these matrices to Fisher's Z transformation.

#### Clustering analysis

Given that DPD patients possess intact reality testing abilities, we hypothesized that distinct states are consistently present across both groups (DPD and HC) and conditions (pre-tDCS and post-tDCS), with variations limited only to dynamics. Consequently, we utilized the K-means algorithm to segment the fMRI sequences from both DPD and HC subjects into distinct states, thereby capturing the underlying patterns of dFNC [48]. We utilize the elbow point criterion to establish the optimal number of clusters (states) [49]. To visually represent the segregation of connectivity states into distinct clusters, we plotted each state in a reduced two-dimensional space using Principal Component Analysis (PCA) for simplification and visualization purposes. The states were color-coded according to their corresponding cluster assignment, facilitating an intuitive visualization of their distribution and separation. The resulting clusters are illustrated in Fig. S1. Following this, we calculate the mean correlation matrix for each state, shedding light on the variations in FC across other states. Simultaneously, we measure the mean dwell time within each state, the frequency of each state's occurrence, and the transitions between states for every subject.

Utilizing the K-means clustering results, we assign different state labels to each time window for each subject in every group (DPD group and HC group). This allows us to obtain dynamic state parameters, including the count (the number of occurrences of a state in a subject), the mean dwell time (the average duration that a state persists), and the number of transitions (the frequency of state changes in a subject). We then compare the state parameters between the DPD and the HC groups using an ANACOVA test, controlling for variables such as sex, age, education, and head framewise displacement.

#### Correlation analysis

We conducted a partial Spearman correlation analysis to examine the relationship between the total score on the CDS and the average duration in each state. In this analysis, we controlled sex, age, years of education, and head movement parameters. A significance level of *P* < 0.05 indicates a statistically significant correlation. Notably, our correlation analysis was conducted as an exploratory study. The primary goal was to identify potential trends and relationships that could inform future research rather than to establish definitive statistical significance. Given the novel nature of this investigation and the limited sample size, applying multiple comparison corrections could have increased the risk of Type II errors, potentially obscuring meaningful findings. Therefore, we chose not to apply multiple comparison corrections in this context, aligning with other exploration studies [50, 51].

#### Pre- and post-tDCS analysis

We applied the K-means model from the Clustering analysis section to pre- and post-tDCS fMRI scan data for post-tDCS assessments. By using this approach to both pre-tDCS, and post-tDCS data, and HC data, we could compare changes in state parameters. Specifically, due to the limited sample size, we provide descriptive statistics for positive-tDCS, negative-tDCS, and HC in pre-tDCS and HC data.

## Results

We recruited 92 DPD subjects, but eight subjects were excluded (one for gross morphological anomalies, three for poor quality control score, three for max head motion > 3 mm, and one for relative RMS > 0.2 mm). Thus, we finally analyzed 84 samples (22 female, 59 unmedicated) with a diagnosis of DPD. And 67 HCs were recruited, matched by age, education duration and sex. For a detailed list of the brain regions involved in each component, see Supplementary Information (SI 1 Clinical information of participants).

### FPN, SMN, and DMN of DPD identified from group ICA

**Fig. 3:**
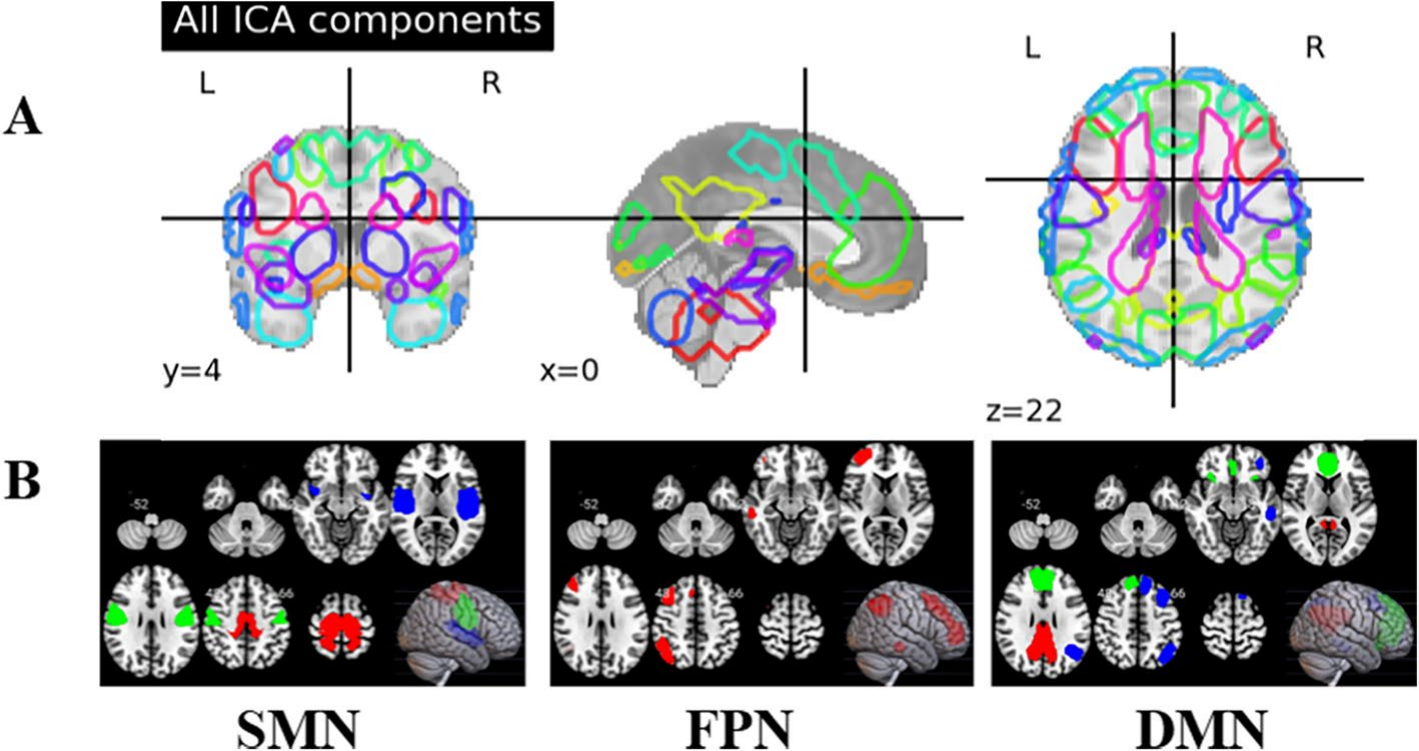
Our DPD atlas. **A** All ICA components; (**B**) Components in SMN, FPN, DMN. The different colors represent different components in the network. Component 1 is characterized by red, Component 2 by green, and Component 3 by blue. Abbreviation: ICA, Independent Component Analysis; SMN, Sensorimotor Network; FPN, Frontoparietal Network; DMN, Default Mode Network

The results of the group ICA are depicted in Fig. 3. Using CanICA, 30 components were identified in our DPD atlas. Among them, three components corresponded to the SMN (as network_2 in the Yeo-7 atlas), one component matched the FPN (Network_6 in the Yeo-7 atlas), and three components aligned with the DMN (Network_7 in the Yeo-7 atlas [46]). For a detailed list of the brain regions involved in each component, see Supplementary Information (SI 2 The brain regions in Components).

### The four states of dFNC identified in DPD

**Fig. 4:**
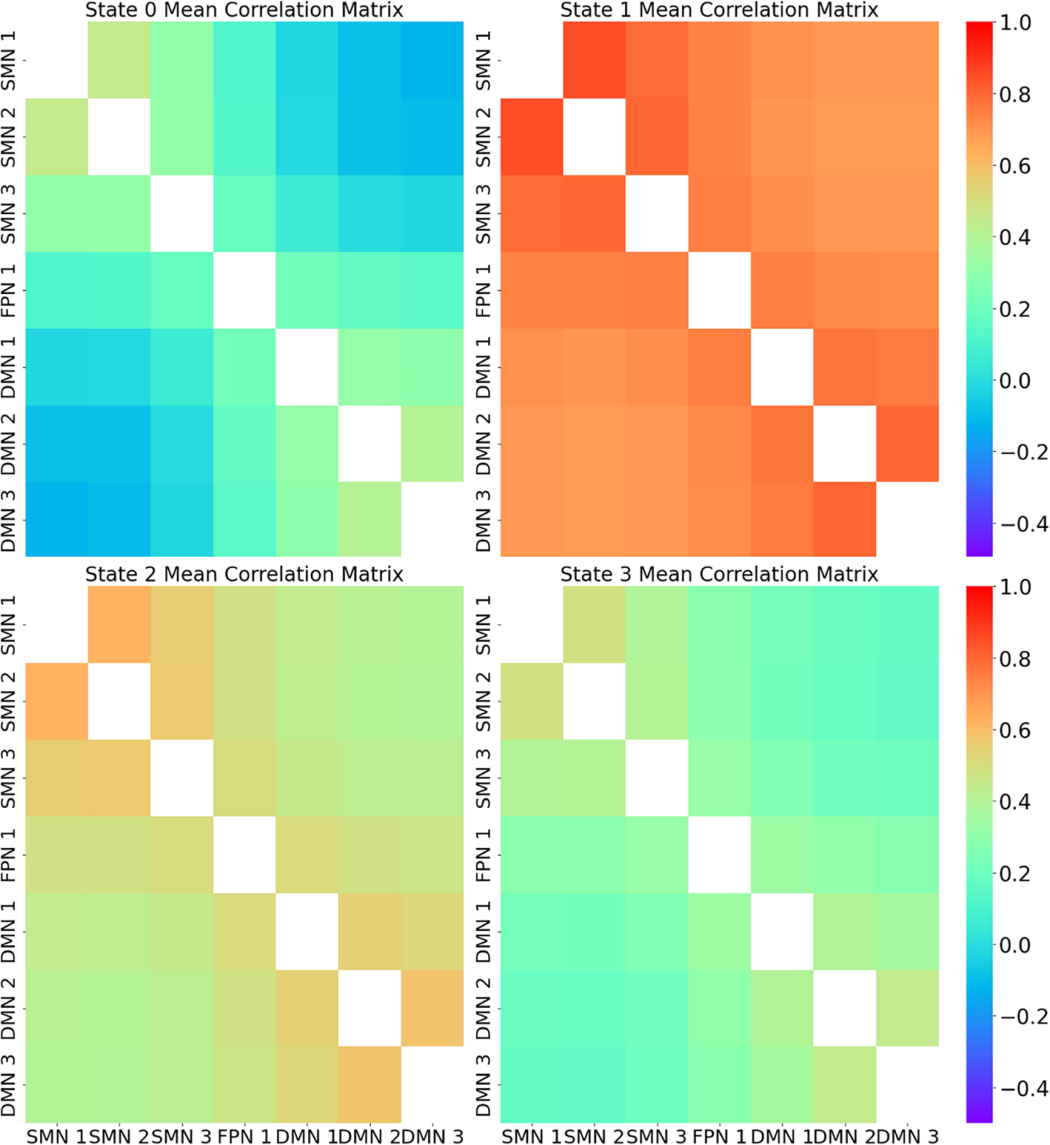
Different states of dFNC. We identified four distinct neural states. Notably, variations in the dwell times of the global cohesive state (State-1) and the intra-network cohesive state (State-2) served as pivotal biomarkers

With the elbow method for K-means clustering, we identified four states of DPD from the dFNC analysis based on our generated DPD atlas (Fig. 4). These clusters are shown in Fig. S1. Varying the number of clusters can significantly affect the outcomes of the analysis. Using fewer clusters might obscure important distinctions between different dynamic states, potentially leading to an oversimplified interpretation of the functional connectivity data. On the other hand, a higher number of clusters could lead to overfitting, where the model captures noise as if it were significant, making the clusters less interpretable and potentially less reproducible across different datasets or similar studies.

In State-0 and State-2, the FC between all components appeared weak. Conversely, State-1 displayed strong FC across all components. State-3 showcased strong intra-network FC within DMN and SMN components. The mean dwell time of State-1, which exhibited a robust active correlation among all components, was different between the DPD group and the HC group (F = 4.10, *P* = 0.045, Table 1), while the dwell time of each state was not different. Detailed information on these parameters is shown in the Supplementary Materials Fig. S2.2–1 to Fig. S2.9.

**Table 1:** ANACOVA result for brain dynamics between DPD and HC group

Variable	DPD (*N* = 84)	HC (*N* = 67)	Sum sq	F-statistic	PR(> F)
State-0 mDT	7.99 ± 5.28	7.07 ± 3.93	44.075	2.54	0.113
State-1 mDT	2.65 ± 2.95	2.85 ± 1.96	16.649	4.945	0.028*
State-2 mDT	3.29 ± 0.94	3.21 ± 0.78	0.01	0.019	0.89
State-3 mDT	4.71 ± 1.62	4.95 ± 1.77	0.383	0.169	0.682
State-0	40.43 ± 27.39	36.46 ± 23.26	2057.314	3.282	0.072
State-1	14.24 ± 19.56	14.73 ± 17.01	210.179	0.978	0.324
State-2	36.89 ± 19.21	37.45 ± 15.28	187.711	0.622	0.432
State-3	50.44 ± 17.71	53.36 ± 17.99	150.245	0.475	0.492
Transitions number	31.67 ± 9.91	31.96 ± 7.81	55.941	0.715	0.399

ANACOVA test, controlling for variables with sex, age, education, and head framewise displacement. Brain dynamics (Variable): State-0 mean dwell time, State-1 mean dwell time (*p < 0.05), State-2 mean dwell time, State-3 mean dwell time, State-0 frequency of occurrence, State-1 frequency of occurrence, State-2 frequency of occurrence, State-2 frequency of occurrence, State-3 frequency of occurrence, Transitions number

*Abbreviation:* *mDT* Mean dwell time, *DPD* Depersonalization-derealization disorder, *HC* Healthy control

### Correlation analysis between dFNC states and clinical assessments

**Fig. 5:**
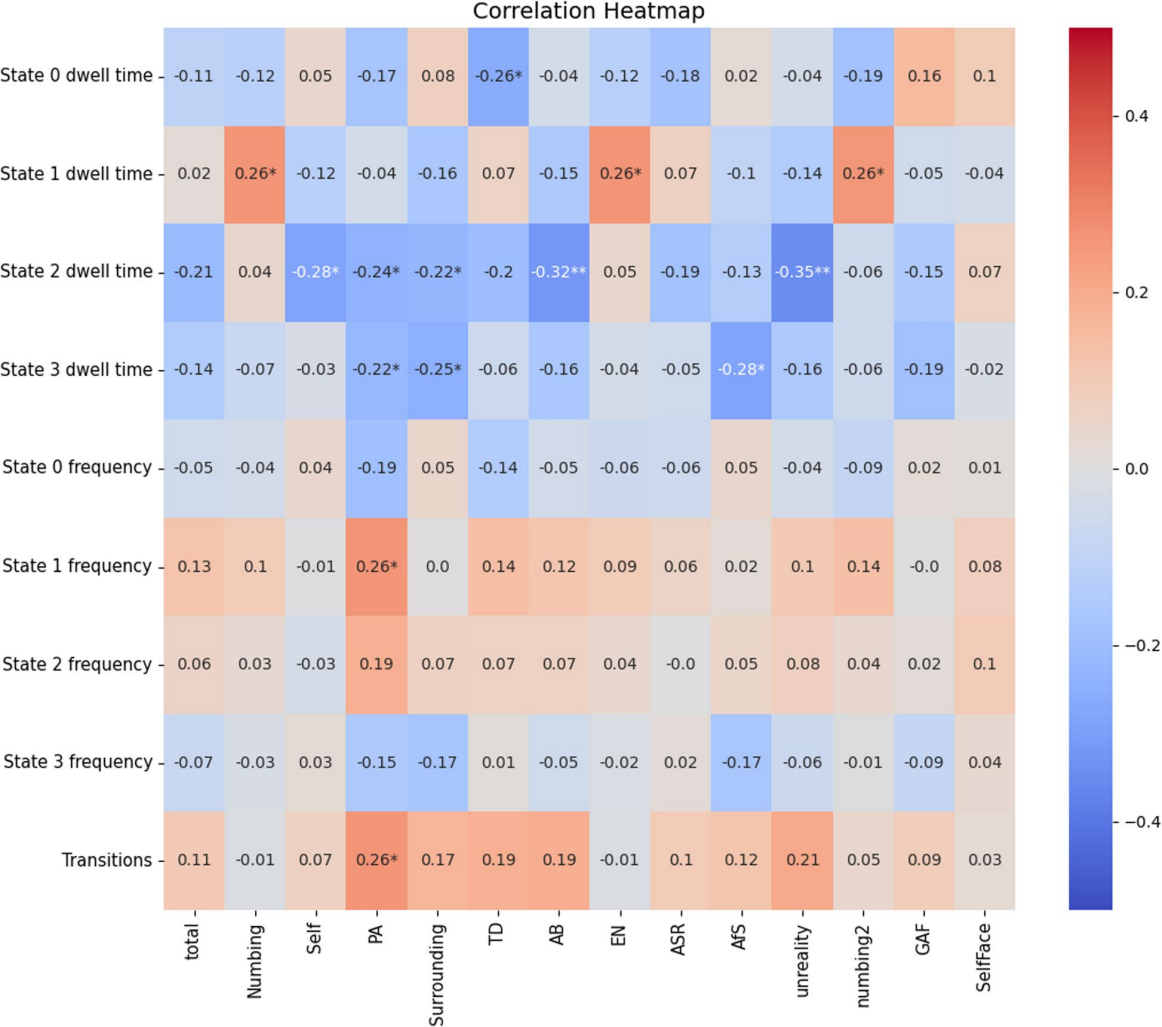
Correlation between DPD neural dynamics and related scales. Total: the total scores of CDS. Five factors of CDS: Numbing, Self (Unreality of Self), PA (Perceptual Alterations), Surrounding (Unreality of Surroundings), TD (Temporal Disintegration); Four factors of CDS: AB (Anomalous Body Experience), EN (Emotional Numbing), ASR (Anomalous Subjective Recall), AfS (Alienation from Surroundings); Two Factors of CDS: Unreality (A sense of unreality and detachment), Numbing2 (Emotional and physical numbing); GAF, global assessment of functioning. The mean dwell time of State-2, an intra-network cohesive state, displayed significant negative correlations with factors such as self (*p* < 0.05), PA (*p* < 0.05), surrounding (*p* < 0.05), AB (*p* < 0.01), and unreality (*p* < 0.01)

Multiple negative associations were observed when analyzing the correlation between the brain dynamics of DPD patients and related scales. Specifically, the mean dwell time of State-2, an intra-network cohesive state, displayed significant negative correlations with factors such as self, PA, surrounding, AB, and unreality (Fig. 5).

### The verification of the dFNC states through tDCS intervention

Due to the limited sample size, we performed only descriptive statistics without hypothesis testing at the group level. Notably, tDCS responders (2.85 ± 1.60) exhibited higher State 1 mean dwell times than HCs (2.85 ± 1.51), consistent with our overall group-level findings. Conversely, non-responders (1.53 ± 0.45) showed lower State 1 mean dwell times than HCs, which may partly explain their lack of response to tDCS treatment. These results are illustrated in Fig. S2.3–1 to Fig. S2.3–9.

A post-tDCS analysis demonstrated a notable change in the mean dwell time for State-2 in responders to tDCS intervention rather than in non-responders (Before: 2.941 ± 1.258, after: 3.477 ± 0.866, *t*-statistic = 4.506, *P* = 0.046, Table 2), while the mean dwell time for State-3 (Before: 3.141 ± 0.70, after: 3.524 ± 1.008, *t*-statistic = 3.086, *P* = 0.022, Table 2) and the number of State-0 (Before: 44.714 ± 30.136, after: 28.857 ± 17.343, *t*-statistic = -4.967, *P* = 0.003, Table 2) changed in non-responders. In both groups, there were no significant side effects were observed, except for the sensation of tDCS stimulation itself. This result is consistent with the findings from the earlier part of this study, where we discovered a correlation between mean dwell time in State-2 and the ratings on the DPD-related clinical assessment.

**Table 2:** Paired t-test of tDCS intervention

	Responders (*N* = 3)		Non-responders(*N* = 7)	
Dependent variable	*t*-statistic	*p*-value	*t*-statistic	*p*-value
State-0 mean dwell time	-0.748	0.533	-0.357	0.736
State-1 mean dwell time	0.997	0.501	1.161	0.298
State-2 mean dwell time	4.506	0.046*	0.529	0.616
State-3 mean dwell time	-0.497	0.669	3.086	0.022*
State-0 frequency of occurrence	0.072	0.949	-4.967	0.003*
State-1 frequency of occurrence	-0.472	0.683	2.231	0.067
State-2 frequency of occurrence	0.675	0.569	1.153	0.293
State-3 frequency of occurrence	-0.072	0.949	1.836	0.116
Transitions number	0.833	0.492	0.094	0.928

Brain dynamics (dependent variable): State-0 mean dwell time, State-1 mean dwell time, State-2 mean dwell time (*p < 0.05 in responders), State-3 mean dwell time (*p < 0.05 in non-responders), State-0 frequency of occurrence (*p < 0.05 in non-responders), State-1 frequency of occurrence, State-2 frequency of occurrence, State-2 frequency of occurrence, State-3 frequency of occurrence, Transitions number

## Discussion

In this paper, we explore the brain dynamics of DPD through dFNC analysis. First, we generated a new comprehensive disease atlas for DPD, where the network atlas in DPD showed lateralized. Second, we found the brain dynamics of active states play a significant role in the DPD mechanism. Notably, the dwell time of the global cohesive state (State-1) emerges as a critical factor distinguishing DPD from HC. Meanwhile, the dwell time of the intra-network cohesive state (State-2) is associated with various DPD symptoms. Third, our post-tDCS analysis revealed that tDCS significantly influences the dwell time of State-2, thus demonstrating our symptom-related observations.

Contrasting with the previous work mentioned [7], our study first employs FC analysis and delves into brain dynamics with multiple analysis methods such as ICA and dFNC. Second, our considerably larger sample size makes our findings more considerable. Third, we adopt the effect of tDCS interventions to understand how treatments influence DPD brain dynamics, which verifies our clinical findings from clinical practice.

### Comprehensive disease FC atlas for DPD

We have successfully generated a comprehensive disease FC atlas for DPD for the first time, significantly advancing our understanding of this complex condition. We found the brain regions in these networks were not aligned with the healthy peoples’ atlas (Yeo-7 [52]). The SMN in the DPD atlas was similar to the Yeo-7 network, while the FPN and DMN seemed to be lateralized. In our previous research [53], we found that patients with DPD demonstrated higher fractional anisotropy (FA) in the right corpus callosum (CC), and posterior corona radiate (CR) compared to HCs. The CC is a structure in the brain that connects the left and right hemispheres through around 180 million transcallosal fibers. It is responsible for monitoring the integration of information between the hemispheres, as well as controlling sensory, motor, and cognitive functions [54]. When the fibers in the genu and body of the CC have higher FA, it indicates abnormal information exchange between the prefrontal lobe and sensory areas of the brain abnormal information exchange between the prefrontal lobe and sensory areas of the brain is indicated [53]. And the network atlas in DPD shown lateralized seems to support our previous findings [53]. These results suggest that lateralization of the brain for DPD may contribute to DPD’s possible pathomechanisms.

In neuroimaging studies, the choice of brain atlas can significantly influence the results, particularly regarding the identified brain networks and dynamic states. For instance, the choice of atlas may influence the statistical significance and characterization of dFNC states. The AAL atlas [55], for example, is widely used and divides the brain based on anatomical landmarks. In contrast, the DPD atlas is designed to emphasize functional connectivity relevant to DPD. These differences in regional definitions and the specificity of the brain areas involved can lead to variations in the identified networks and dynamic states. The DPD atlas is tailored to reflect the neural alterations observed in DPD, offering a more precise framework for our analysis. This atlas allows for a focused examination of brain regions that are particularly implicated in DPD, thereby enhancing the accuracy and relevance of our findings. On the other hand, using a more general atlas like AAL might lead to broader but less specific findings, potentially overlooking subtle but critical connectivity patterns associated with DPD. Therefore, the conclusions regarding the neural mechanisms underlying DPD might differ depending on the atlas used. By choosing the DPD atlas, we aim to ensure that our results are as relevant and specific as possible to the disorder in question, thereby providing deeper insights into its neural underpinnings.

Thus, we chose the DPD atlas for this study due to its high relevance and specificity for DPD. And due to our hypothesis focused on exploring the differences in dFNC, rather than static network structures. We chose to compare the different states between groups rather than directly compare components or networks between the groups. This approach is consistent with other studies in the field [56, 57], which often prioritize investigating network dynamics and their implications for cognitive and clinical outcomes rather than static network configurations.

### The differences in brain dynamics between DPD and HC

Our dFNC analysis revealed pronounced discrepancies in brain dynamics between the HC group and the DPD group. Notably, we identified distinct patterns in the dwell time of the global cohesive state, which may suggest that brain dynamics could be the potential biomarker for DPD diagnosis.

Our dFNC analysis discerned four distinct states associated with DPD. In State-0 and State-2, FC between all components seemed weak. In contrast, state 1 exhibited strong FC across all components, while state 3 demonstrated a pronounced intra-network FC within DMN and SMN components. A key observation was the disparity in the dwell time of the global cohesive state (State-1), which appears to be a critical factor differentiating DPD from HC. The global cohesive state, characterized by robust FC among the SMN, FPN, and DMN, represents heightened synchronized activity across these major brain networks [58]. The SMN is responsible for sensory and motor processing [59], contributing to the bodily self [60]; the FPN governs executive functions like attention and working memory [61], while the DMN is involved in self-referential thoughts and daydreaming [62]. A disparity in the dwell time of this state between DPD and HC suggests a different pattern of inter-network communication in DPD patients. This difference might indicate that DPD patients have altered integration or co-activation patterns among sensory processing, executive functions, and self-referential thoughts. Such a shift could potentially contribute to the dissociative experiences of DPD. In other words, the heightened synchronization across these networks might underlie the detachment from reality and self that DPD patients report. The prolonged dwell time in this state for DPD patients compared to HCs might suggest a greater tendency for them to stay in a hyper-connected neural state, which could correlate with the intensity or frequency of their DPD symptoms.

The observed disparity in the dwell time of the State-1 between the DPD group and the HC group suggests that DPD individuals may experience a hyper-connected neural state, a phenomenon that aligns with the predictive coding framework. This framework proposes that depersonalization and derealization symptoms can be individually manipulated by altering the relative precision of interoceptive predictions driven by exteroceptive, proprioceptive, and interoceptive sensory modalities. According to Gatus et al. [63], activation of the insula (part of the SMN) and ACC (part of the DMN) activation together serve to integrate perception and physiological responses. We could potentially propose that in healthy people, the self-reference of any given signal is formed by iteratively comparing the signals with the predicted self-reference of that signal (through interoceptive- to Mental-self-processing) and by dynamically updating the predictions to minimize prediction errors, resulting in both bottom-up and top-down modulations [31]. DPD patients stay in a hyper-connected state for more time, then the level of self-processing could be impaired, which leads to altered interceptive prediction and ultimately results in symptoms of numbing.

Although these comparisons did not yield statistically significant differences, we believe this result is due to several factors. The variability and complexity of brain functional connectivity, combined with a relatively small sample size, may obscure subtle differences between the groups. Furthermore, the exploratory nature of our study was aimed at identifying potential patterns and trends rather than achieving definitive statistical comparisons.

Using the Yeo-7 atlas and ICA with 30 components was intended to capture broad and complex network patterns, which might not be sensitive enough to detect finer-grained differences. Nonetheless, the overall patterns observed in our analyses provide valuable insights into the brain dynamics of DPD patients.

### The correlation of brain dynamics with DPD symptomatology

When correlating brain state dynamics with CDS scores, we found that the dwell time of the intra-network cohesive state for both DMN and SMN (State 2) appears to negatively correlate with various DPD-related symptom scores. Interestingly, States-1 and State-2 illuminate contrasting aspects concerning the differences between DPD and HC and the relationship of brain dynamics with DPD experiences. State-1, representing the global cohesive state, reveals fundamental neural differences between DPD patients and HCs. It captures an overarching neural signature of DPD but might not directly correlate with specific real-time disorder experiences. This distinction suggests it embodies a generalized predisposition towards DPD rather than a direct measure of symptom intensity. Conversely, State-2, which showcases activity within both DMN and SMN, correlates more closely with transient DPD experiences. In summary, while State-1 offers a broader perspective on the inherent neural distinctions between DPD patients and HCs, State-2 delves into the dynamic neural processes associated with the immediate expression of DPD symptoms, considering both introspective and sensory experiences. These findings further suggest that brain dynamics could be the potential biomarkers for DPD progress monitoring.

The DMN is traditionally associated with self-referential activities [62], introspection, and daydreaming. In the context of DPD, the negative correlation with DMN activity suggests that increased activity within this network might serve as a compensatory mechanism or protective factor, potentially mitigating the severity of DPD symptoms. In simpler terms, when the DMN is more active or internally synchronized (as indicated by the longer dwell times in state 2), it may counterbalance or reduce the intensity of dissociative and unreality experiences that DPD patients undergo. Conversely, the SMN, primarily involved in sensory processing and motor functions [64], also exhibits altered activity in DPD patients [65]. While its specific correlation with DPD symptoms requires more in-depth analysis, it can be hypothesized that disruptions or heightened activity in SMN might relate to sensory or perceptual disturbances often reported in DPD [65].

Previous research also supports our findings of the relationship between DMN/SMN activities and DPD symptoms. Parts of DMN (i.e., right ACC and mPFC) were activated when DPD sufferers differentiate between self and others [17]. Notably, the magnitude of Depersonalization correlates with activations in mPFC, among other areas. Reinforcing this, our preceding investigation [34] emphasized the instrumental role of mPFC in self-referential processes and its potential involvement in DPD pathologies [29]. Moreover, parts of SMN(i.e. insula) were identified as a linkage between symptom alleviation in DPD, with diminished emotional resonance tethered to a subdued insula response [22]. Many studies have positioned the insula as pivotal to interoceptive awareness, our innate self-sensing faculty [66–71].

### The verification of dFNC states through tDCS intervention

In our exploration of DPD, the application of tDCS played a pivotal role in verifying the dFNC states we identified, which offered a unique perspective on how these states respond to neuro-modulatory interventions [72]. The use of tDCS adds significant credibility to our findings. It not only reinforces the identified brain dynamics as key biomarkers in DPD but also suggests that these states are modifiable, emphasizing the need for targeted therapeutic interventions.

In detail, our analysis revealed distinct responses to tDCS intervention in DPD patients, categorized as responders and non-responders, with no significant side effects observed. We investigated the differences in dynamic states between DPD patients and HCs before tDCS treatment. Our analysis revealed distinctions between responders, non-responders, and HCs. The dwell time of State 1 in responders was higher than HCs, consistent with our primary results. While that in the non-responders was lower than in the HCs. This finding suggests that these patients' neural dynamics may differ, potentially explaining the lack of therapeutic effect observed in this subgroup. These results underscore the importance of considering individual differences in neural dynamics when evaluating the efficacy of tDCS for treating DPD. The observed variations in dynamic states highlight the potential for personalized treatment approaches, where identifying specific neural profiles could guide the selection of patients who are more likely to benefit from tDCS. However, it is important to note that the small sample size in our study may introduce bias, and further research with larger cohorts is necessary to validate these findings and explore their implications for personalized treatment strategies in DPD.

In responders, tDCS notably influenced the dwell time of State-2, the intra-network cohesive state. This alteration underscores the potential of tDCS in modifying the activity within the DMN and SMN. These networks are deeply involved in the self-referential and sensory processing dysfunctions in DPD, suggesting that tDCS might recalibrate internal synchronization in these networks, thereby alleviating DPD symptoms. The change of State-2 dwell time in responders reaffirms our earlier findings, indicating that increased activity within these networks, particularly the DMN, might serve as a protective mechanism in DPD. The longer dwell times in State-2 might help counterbalance or reduce the intensity of dissociative and unreality experiences in DPD patients. Conversely, non-responders exhibited changes in the dwell time of State-3 and the number of occurrences in State-0 post-tDCS treatment, which might reflect a different mechanism of brain adaptation or resistance to tDCS intervention. These observations for the tDCS effect are particularly intriguing, which imply that while tDCS can alter brain dynamics in DPD, its effectiveness and the nature of these changes can vary significantly among individuals [73].

### Limitation

Whilst our study provides valuable insights, it is not devoid of limitations. Although necessary for ensuring quality, the exclusion of certain data might lead to potential biases [74]. Additionally, the correlational nature of some findings prevents us from establishing causality [75]. The small sample size used in the tDCS intervention may result in low statistical power. It is also worth noting that the exact clinical significance of each dFNC state remains to be fully decoded, an area for future exploration.

## Conclusion

In conclusion, our research suggests the brain dynamics of DPD could be a potential biomarker for diagnosis and symptom analysis, which may be helpful for further clinical research for DPD. We presented a comprehensive disease atlas, found the active state plays a significant role in the DPD mechanism, and demonstrated our symptom-related observations with post-tDCS analysis. Key findings reveal that the active state significantly influences the mechanism of DPD. Notably, variations in the dwell time of the global cohesive state serve as a crucial factor distinguishing DPD from HCs. Furthermore, the dwell time of the intra-network cohesive state correlates with various DPD symptoms, as evidenced by post-tDCS treatment observations. These insights advance our knowledge of DPD mechanisms and emphasize the promising therapeutic potential of interventions such as tDCS, which also benefit finding innovative management approaches for this complex disorder.

While our study benefits from a larger sample size compared to previous research [7], future work could still gain from analyzing even more extensive data to produce findings that are more representative of the real DPD population. Additional, we consider incorporating state-of-the-art explainable deep learning techniques for treatment prediction in DPD mechanism research, aiming for a more objective understanding of the DPD mechanism that surpasses human selection bias [76].

## Supplementary Information

Supplementary Material 1.

Supplementary Material 2.

## Data Availability

The data that support the findings of this study are available from the Beijing Anding Hospital but restrictions apply to the availability of these data, which were used under license for the current study, and so are not publicly available. Data is however available from the authors, Sisi Zheng (email address: zhengsisi@ccmu.edu.cn), and Hongxiao Jia (email address: jhxlj@ccmu.edu.cn), upon reasonable request and with permission of the Beijing Anding Hospital. Additionally, the atlas generated during our research is included as supplementary materials in this paper, making it publicly available for further DPD research.

## Abbreviations

DPD: Depersonalization-Derealization Disorder
FPN: Frontoparietal Network
SMN: Sensorimotor Network
DMN: Default Mode Network
FC: Functional Connectivity
dFNC: Dynamic Functional Network Connectivity
ACC: Anterior Cingulate
mPFC: Medial Prefrontal Cortex
dlPFC: Dorsolateral Prefrontal Cortex
HC: Healthy Control
tDCS: Transcranial Direct Current Stimulation
fMRI: Functional Magnetic Resonance Imaging
ICA: Independent Component Analysis
