# Supplementary material for: Unraveling the brain dynamics of Depersonalization-Derealization Disorder: a dynamic functional network connectivity analysis
Source: BMC Psychiatry. 2024 Oct 14;24:685. doi: 10.1186/s12888-024-06096-1 (PMC11475637; doi:10.1186/s12888-024-06096-1)
Supplement: Supplementary file 2 — Supplementary Material 2. [file 12888_2024_6096_MOESM2_ESM.docx]

**Supplementary Materials**

[Tables 2](#_Toc27275)

[SI 1 Clinical information of participants 2](#_Toc31899)

[SI 2 The brain regions in Components 3](#_Toc4255)

[SI 3 Descriptive Statistics for Brain Dynamics Between Pre-tDCS and Post-tDCS Groups 4](#_Toc28588)

[Figures 4](#_Toc21548)

[1. Cluster Visualization using PCA 4](#_Toc26852)

[Figure S1 Cluster Visualization using PCA 6](#_Toc20101)

[2. Violin plots 6](#_Toc13047)

[2.1 DPD vs HC 6](#_Toc909)

[Figure S2.1-1 Differences in State 0 between DPD and HC group. 6](#_Toc7737)

[Figure S2.1-2 Differences in State 0 mean dwell time between DPD and HC group. 7](#_Toc22111)

[Figure S2.1-3 Differences in State 1 between DPD and HC group. 7](#_Toc19741)

[Figure S2.1-4 Differences in State 1 dwell time between DPD and HC group. 8](#_Toc9281)

[Figure S2.1-5 Differences in State 2 between DPD and HC group. 8](#_Toc25074)

[Figure S2.1-6 Differences in State 2 dwell time between DPD and HC group. 9](#_Toc16374)

[Figure S2.1-7 Differences in State 3 between DPD and HC group. 9](#_Toc32593)

[Figure S2.1-8 Differences in State 3 dwell time between DPD and HC group. 10](#_Toc6391)

[Figure S2.1-9 Differences in transition number between DPD and HC group. 10](#_Toc22799)

[2.2 Pre-tDCS vs post-tDCS 10](#_Toc29978)

[Figure S2.2-1 Differences in State 0 between pre-tDCS and post-tDCS. 11](#_Toc16821)

[Figure S2.2-2 Differences in State 0 dwell time between pre-tDCS and post-tDCS. 11](#_Toc6306)

[Figure S2.2-3 Differences in State 1 between pre-tDCS and post-tDCS. 12](#_Toc16589)

[Figure S2.2-4 Differences in State 1 dwell time between pre-tDCS and post-tDCS. 12](#_Toc22501)

[Figure S2.2-5 Differences in State 2 between pre-tDCS and post-tDCS. 13](#_Toc24709)

[Figure S2.2-6 Differences in State 2 dwell time between pre-tDCS and post-tDCS. 13](#_Toc9944)

[Figure S2.2-7 Differences in State 3 between pre-tDCS and post-tDCS. 14](#_Toc5495)

[Figure S2.2-8 Differences in State 3 dwell time between pre-tDCS and post-tDCS. 14](#_Toc7748)

[Figure S2.2-9 Differences in transition number between pre-tDCS and post-tDCS. 15](#_Toc31122)

[2.3 Pre-tDCS vs HC 15](#_Toc27512)

[Figure S2.3-1 Differences in State 0 among responders, non-responders, and HCs in pre-tDCS data. 15](#_Toc7039)

[Figure S2.3-2 Differences in State 0 dwell time among responders, non-responders, and HCs in pre-tDCS data. 16](#_Toc1714)

[Figure S2.3-3 Differences in State 1 among responders, non-responders, and HCs in pre-tDCS data. 16](#_Toc28147)

[Figure S2.3-4 Differences in State 1 dwell time among responders, non-responders, and HCs in pre-tDCS data. 17](#_Toc11664)

[Figure S2.3-5 Differences in State 2 among responders, non-responders, and HCs in pre-tDCS data. 17](#_Toc32461)

[Figure S2.3-6 Differences in State 2 dwell time among responders, non-responders, and HCs in pre-tDCS data. 18](#_Toc19015)

[Figure S2.3-7 Differences in State 3 among responders, non-responders, and HCs in pre-tDCS data. 18](#_Toc7159)

[Figure S2.3-8 Differences in State 3 dwell time among responders, non-responders, and HCs in pre-tDCS data. 19](#_Toc30078)

[Figure S2.3-9 Differences in transition number among responders, non-responders, and HCs in pre-tDCS data. 19](#_Toc31831)

**Tables**

**SI 1** **Clinical information of participants**

|  | DPD (n=84) | HC (n=67) | χ^2^/U | p value |
| --- | --- | --- | --- | --- |
| Age | 23.0 (20.0,26.5) | 22.0 (19.0,24.5) | 3250 | 0.102 |
| Education Duration | 15.0 (12.0,16.0) | 15.0 (12.5,16.0) | 2459 | 0.213 |
| Sex (Female/male) | 22/63 | 25/42 | 2.292 | 0.13 |
| CDS | 165.85±47.57 |  |  |  |
| Five Factors |  |  |  |  |
| Numbing | 31.50±15.81 |  |  |  |
| Unreality of Self | 39.07±14.21 |  |  |  |
| Perceptual Alterations | 19.14±11.82 |  |  |  |
| Unreality of Surroundings | 14.69±5.38 |  |  |  |
| Temporal Disintegration | 22.16±9.94 |  |  |  |
| Four Factors |  |  |  |  |
| Anomalous Body Experience | 50.64±19.791 |  |  |  |
| Emotional Numbing | 34.82±15.444 |  |  |  |
| Anomalous Subjective Recall | 25.48±11.980 |  |  |  |
| Alienation from Surroundings | 31.67±7.653 |  |  |  |
| Two Factors |  |  |  |  |
| A Sense of Unreality and Detachment | 73.20±24.73 |  |  |  |
| Emotional and Physical Numbing | 64.77±22.95 |  |  |  |
| GAF | 59.22±9.847 |  |  |  |

Note: one patient’s education duration and 22 patients’ GAF were missed. Abbreviation: HC, healthy control; CDS, Cambridge Depersonalization Scale; GAF, global assessment of functioning.

**SI 2 The brain regions in Components**

| Network | Component | Cluster | Cluster Size | Brain regions |
| --- | --- | --- | --- | --- |
| SMN | 1 | 1 | 2056 | Supp_Motor_Area_R, Paracentral_Lobule_L, Postcentral_R,  Precentral_R, Supp_Motor_Area_L, Paracentral_Lobule_R，Precentral_L, Postcentral_L, Precuneus_L,Parietal_Sup_R,  Cingulate_Mid_L, Precuneus_R, Parietal_Sup_L |
|  | 2 | 1 | 875 | Precentral_R, Postcentral_R, Rolandic_Oper_R |
|  |  | 2 | 758 | Postcentral_L, Precentral_L,Rolandic_Oper_L, SupraMarginal_L, Insula_L,Parietal_Inf_L, Frontal_Inf_Oper_L |
|  | 3 | 1 | 1084 | Temporal_Sup_L, Insula_L, Rolandic_Oper_L, Heschl_L, SupraMarginal_L,Postcentral_L, Frontal_Inf_Oper_L, Temporal_Pole_Sup_L, Putamen_L |
|  |  | 2 | 946 | Insula_R,Rolandic_Oper_R,Temporal_Sup_R,Heschl_R, Putamen_R, SupraMarginal_R, Frontal_Inf_Oper_R,Temporal_Pole_Sup_R |
| FPN | 1 | 1 | 936 | Frontal_Mid_2_R, Frontal_Sup_2_R, Frontal_Inf_Tri_R, Frontal_Inf_Orb_2_R |
|  |  | 2 | 534 | Angular_R, Parietal_Inf_R, Parietal_Sup_R, SupraMarginal_R, Occipital_Sup_R |
|  |  | 3 | 68 | Temporal_Mid_R, Temporal_Inf_R |
| DMN | 1 | 1 | 1480 | Precuneus_R, Precuneus_L, Cingulate_Mid_L, Cingulate_Post_L, Cingulate_Mid_R,Cingulate_Post_R, Cuneus_L |
|  | 2 | 1 | 1848 | Frontal_Sup_Medial_L,Frontal_Sup_Medial_R,  ACC_pre_L, ACC_pre_R,ACC_sup_L, Frontal_Sup_2_R, ACC_sup_R, Frontal_Sup_2_L, Frontal_Med_Orb_R |
|  | 3 | 1 | 641 | Angular_L, Parietal_Inf_L |
|  |  | 2 | 321 | Frontal_Sup_2_L,Frontal_Sup_Medial_L, Supp_Motor_Area_L |
|  |  | 3 | 279 | Frontal_Mid_2_L |
|  |  | 4 | 182 | Temporal_Mid_L |
|  |  | 5 | 181 | Frontal_Mid_2_L, Frontal_Sup_2_L |

**SI 3 Descriptive Statistics for Brain Dynamics Between Pre-tDCS and Post-tDCS Groups**

| Brain dynamics | Non-responders (N = 7) | | | | Responders (N = 3) | | | |
| --- | --- | --- | --- | --- | --- | --- | --- | --- |
|  | Pre-tDCS | | Post-tDCS | | Pre-tDCS | | Post-tDCS | |
|  | Mean | Std | Mean | Std | Mean | Std | Mean | Std |
| State 0 | 44.71 | 30.14 | 28.86 | 17.34 | 33.00 | 22.11 | 33.33 | 7.09 |
| State 0 mean dwell time | 8.29 | 3.22 | 7.15 | 2.90 | 6.94 | 3.00 | 6.21 | 0.74 |
| State 1 | 15.00 | 25.21 | 14.43 | 10.56 | 18.00 | 26.96 | 12.33 | 6.43 |
| State 1 mean dwell time | 1.54 | 0.69 | 3.18 | 2.77 | 2.85 | 2.27 | 2.60 | 2.09 |
| State 2 | 42.43 | 21.23 | 46.71 | 15.23 | 33.00 | 29.82 | 39.67 | 12.01 |
| State 2 mean dwell time | 3.14 | 0.71 | 3.52 | 1.01 | 2.94 | 1.26 | 3.48 | 0.87 |
| State 3 | 39.86 | 18.60 | 52.00 | 7.90 | 58.00 | 34.12 | 56.67 | 10.26 |
| State 3 mean dwell time | 3.53 | 0.73 | 4.72 | 0.76 | 5.67 | 3.22 | 5.29 | 1.39 |
| num transitions | 36.14 | 10.22 | 33.86 | 6.94 | 28.00 | 10.00 | 33.00 | 5.57 |

Figures

1. Cluster Visualization using PCA


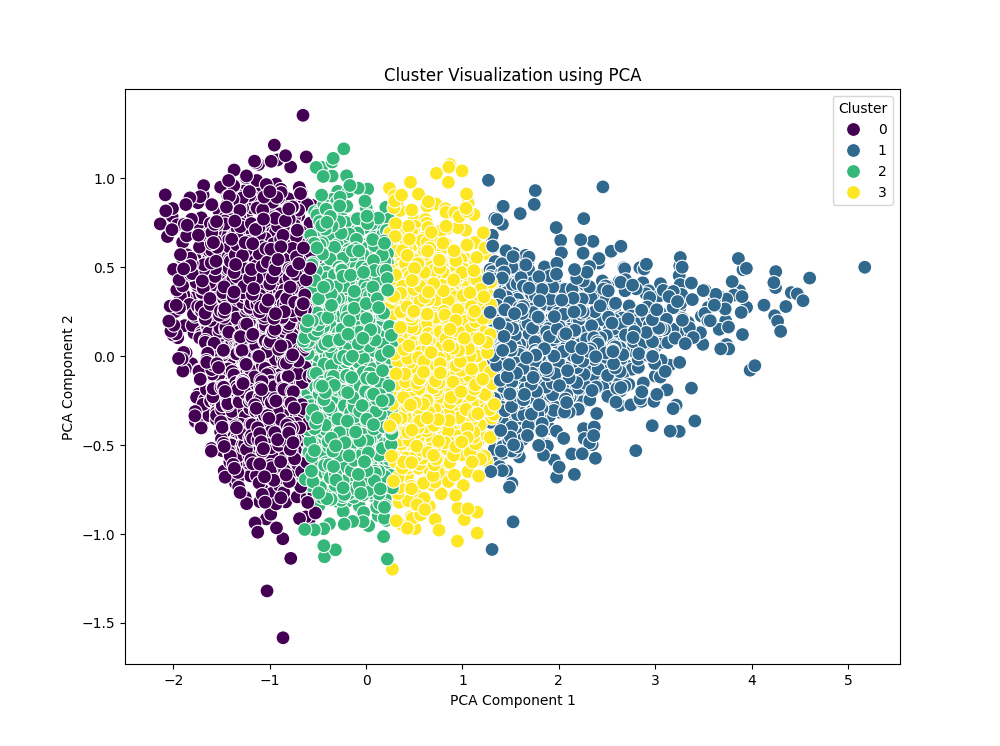


Figure S1. Cluster Visualization using PCA. This scatter plot demonstrates PCA applied to our correlation matrixes, effectively reducing its complexity to two dimensions. It distinctly segments the data into four clusters (0, 1, 2, 3), color-coded by cluster assignment, highlighting their separation and distribution based on K-means clustering.

1. Violin plots
   1. DPD vs HC


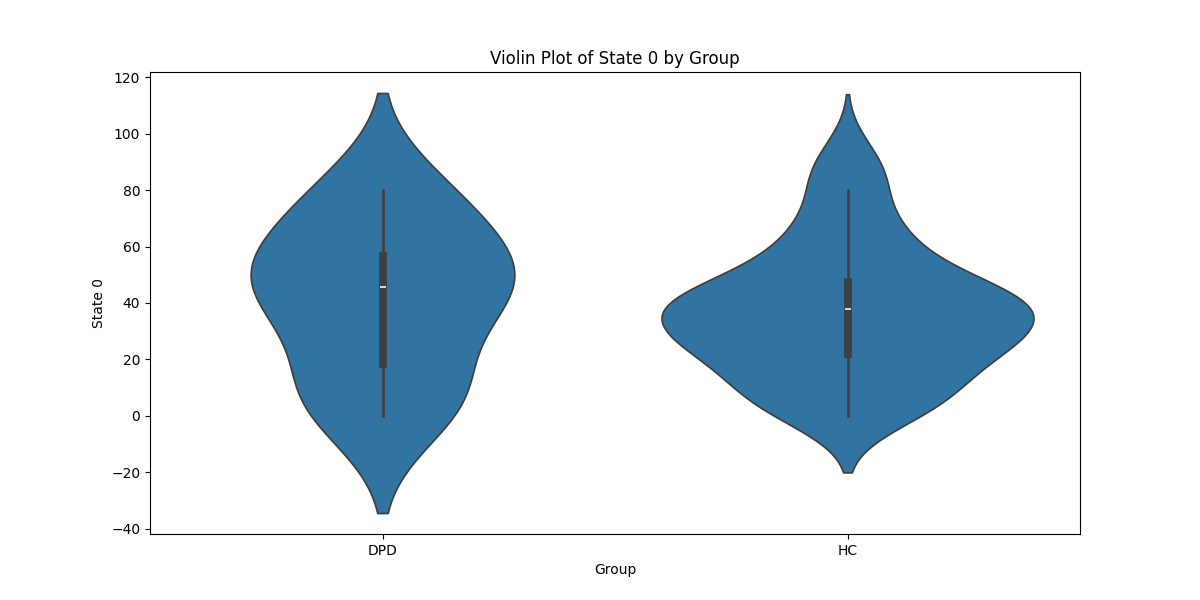


Figure S2.1-1 Differences in State 0 between DPD and HC group.

Abbreviation: DPD, depersonalizaiton-derealization disorder; HC, healthy control


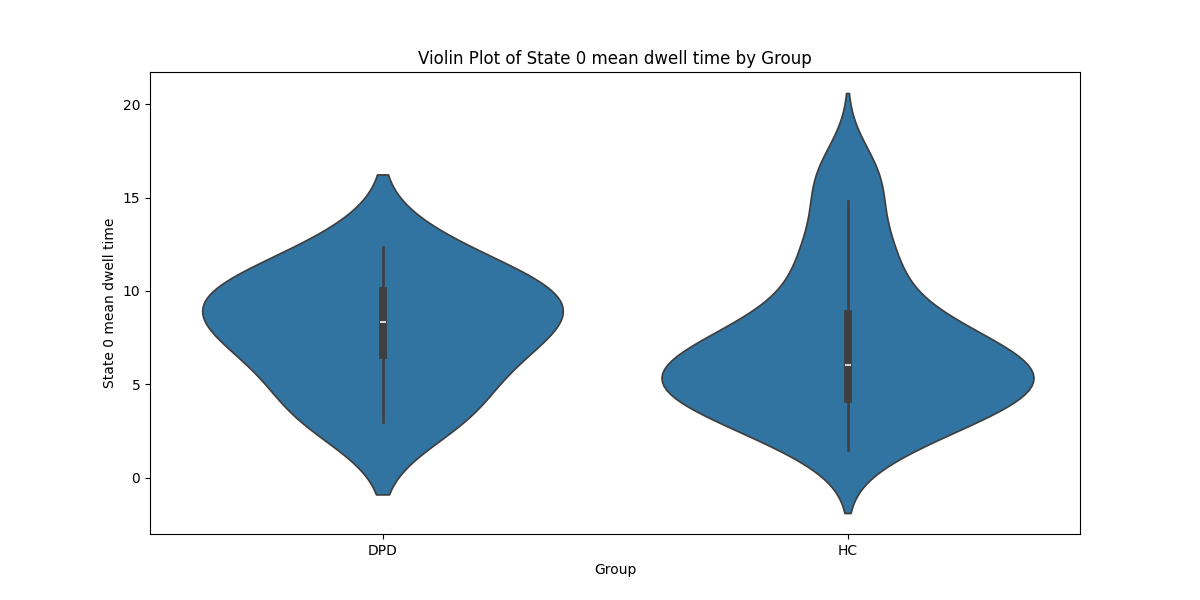


Figure S2.1-2 Differences in State 0 mean dwell time between DPD and HC group.

Abbreviation: DPD, depersonalizaiton-derealization disorder; HC, healthy control


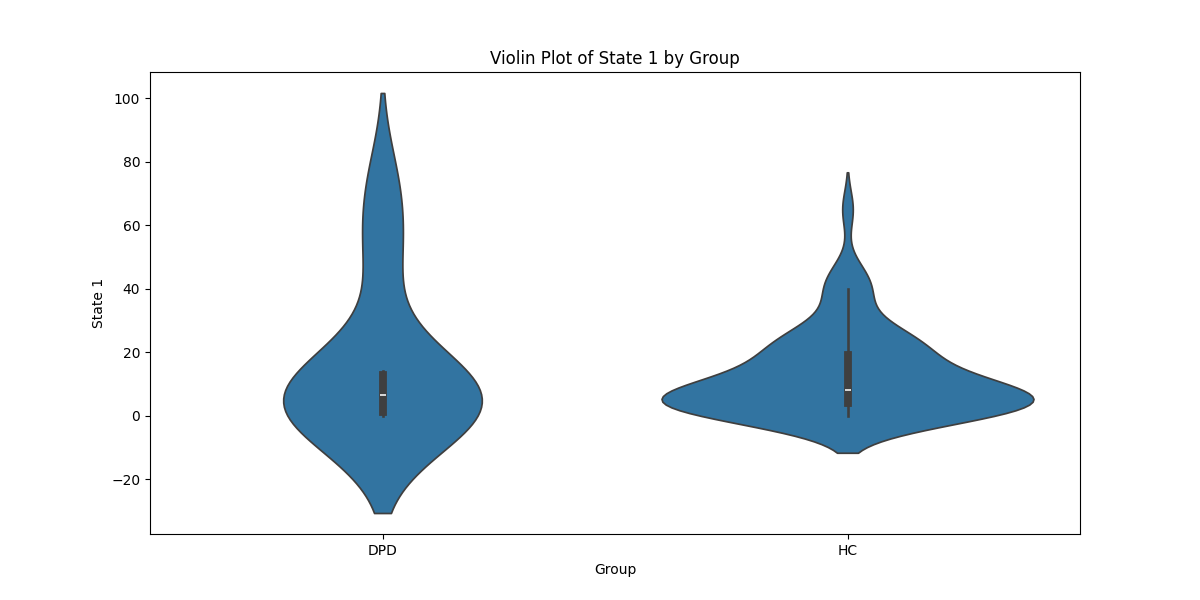


Figure S2.1-3 Differences in State 1 between DPD and HC group.

Abbreviation: DPD, depersonalizaiton-derealization disorder; HC, healthy control


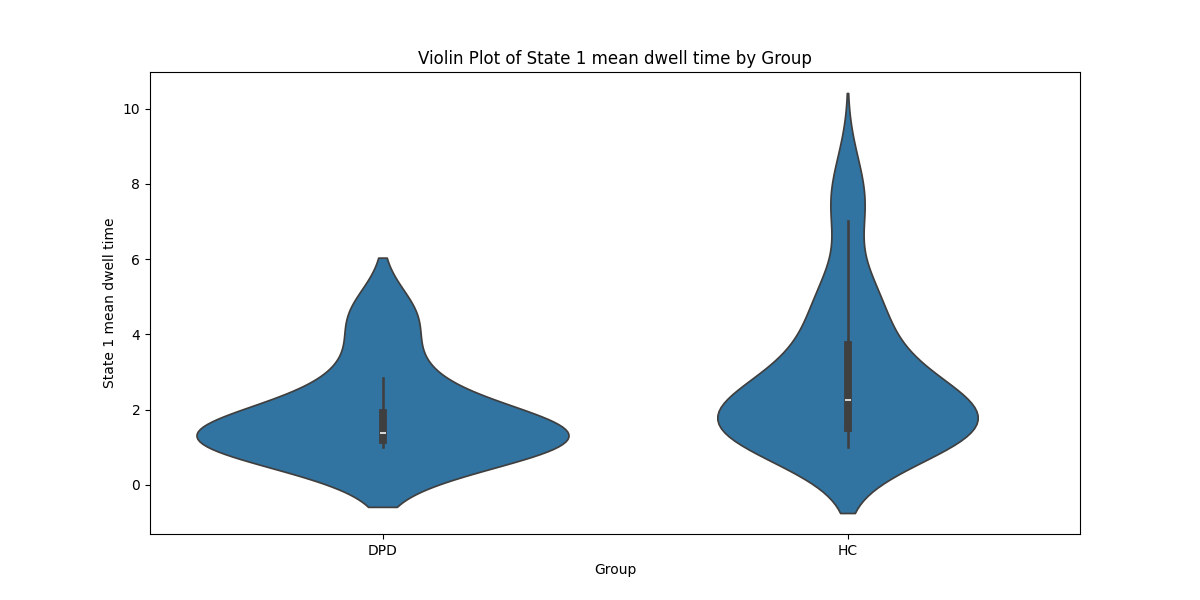


Figure S2.1-4 Differences in State 1 dwell time between DPD and HC group.

Abbreviation: DPD, depersonalizaiton-derealization disorder; HC, healthy control


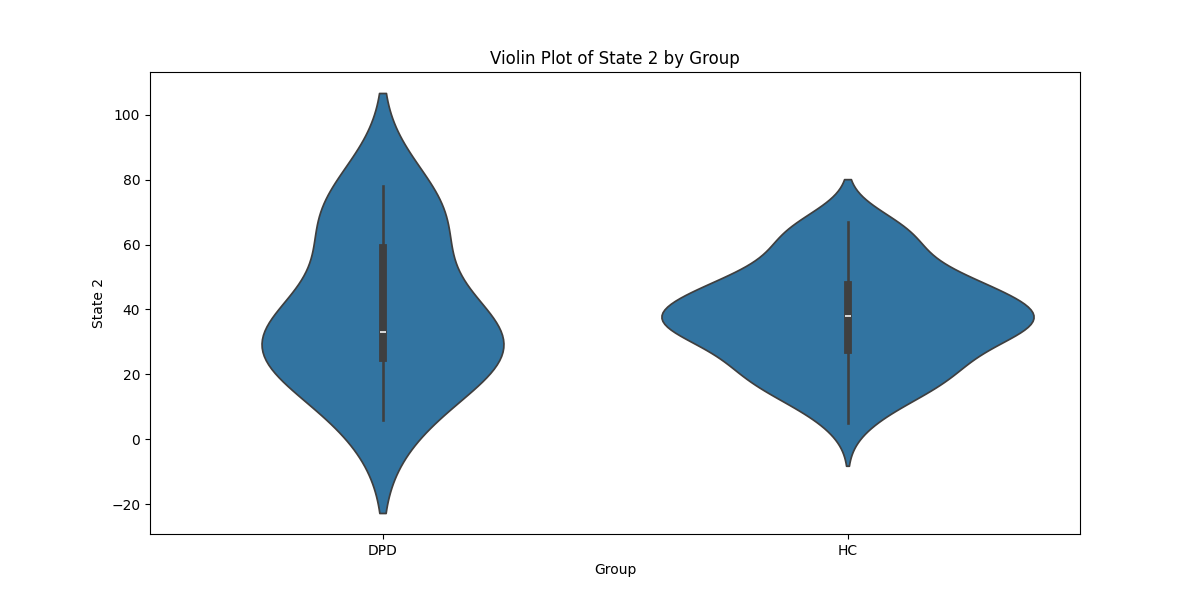


Figure S2.1-5 Differences in State 2 between DPD and HC group.

Abbreviation: DPD, depersonalizaiton-derealization disorder; HC, healthy control


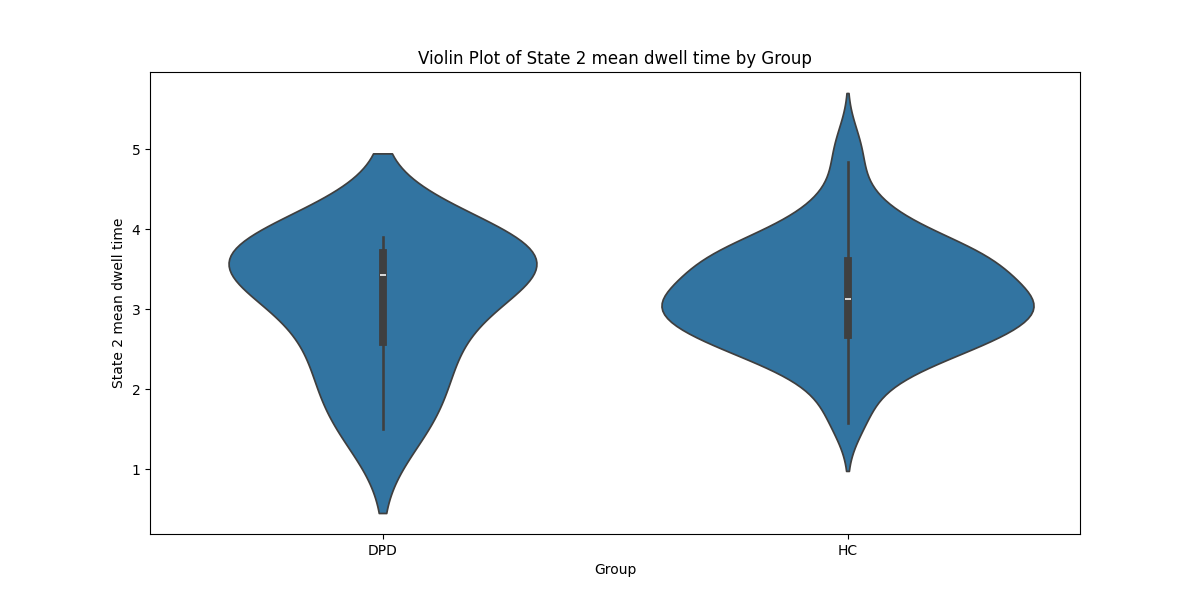


Figure S2.1-6 Differences in State 2 dwell time between DPD and HC group.

Abbreviation: DPD, depersonalizaiton-derealization disorder; HC, healthy control


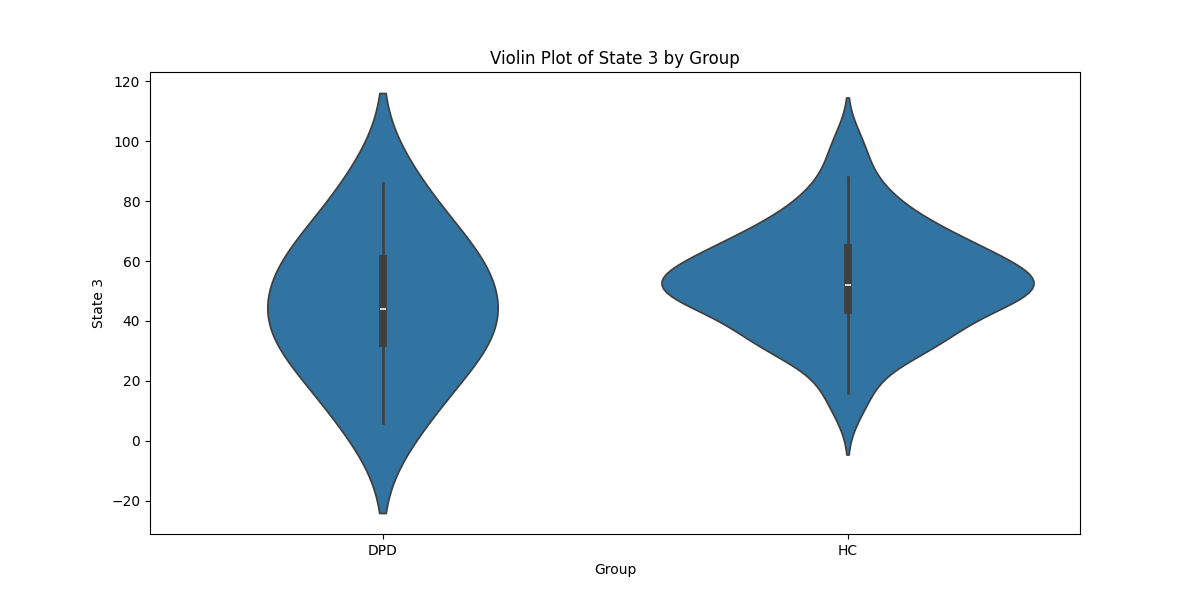


Figure S2.1-7 Differences in State 3 between DPD and HC group.

Abbreviation: DPD, depersonalizaiton-derealization disorder; HC, healthy control


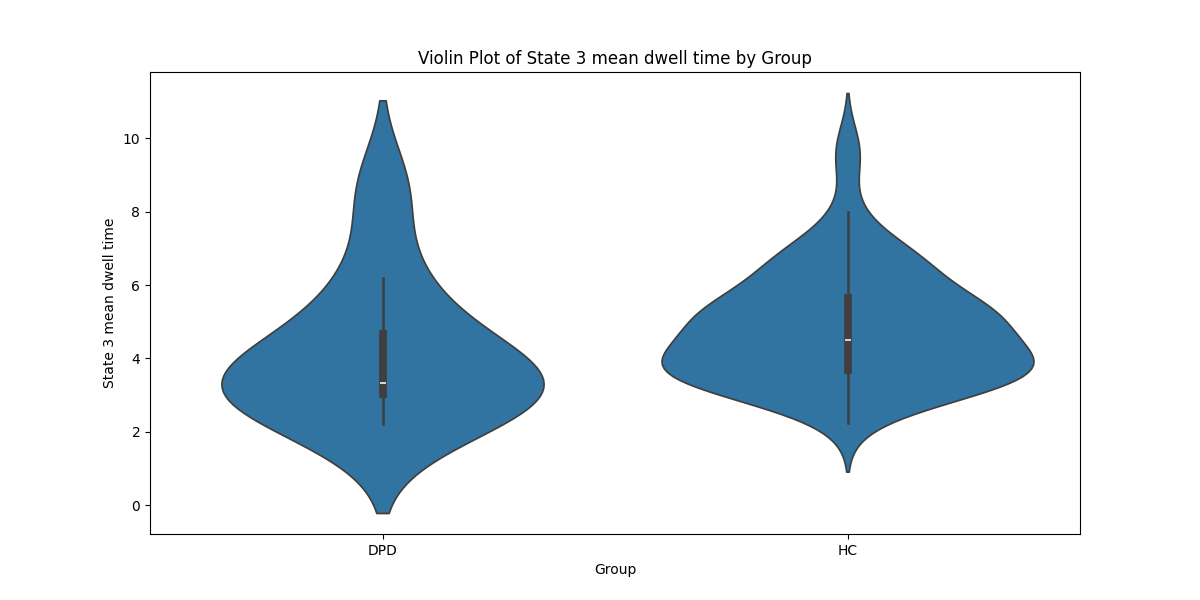


Figure S2.1-8 Differences in State 3 dwell time between DPD and HC group.

Abbreviation: DPD, depersonalizaiton-derealization disorder; HC, healthy control


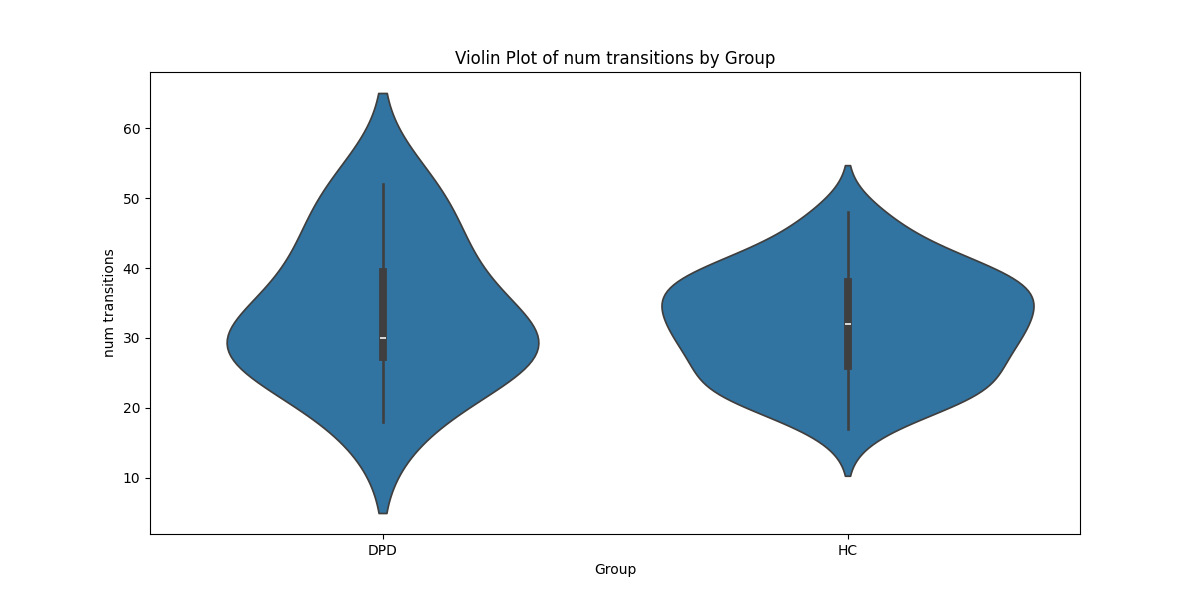


Figure S2.1-9 Differences in transition number between DPD and HC group.

Abbreviation: DPD, depersonalizaiton-derealization disorder; HC, healthy control

- 1. Pre-tDCS vs post-tDCS


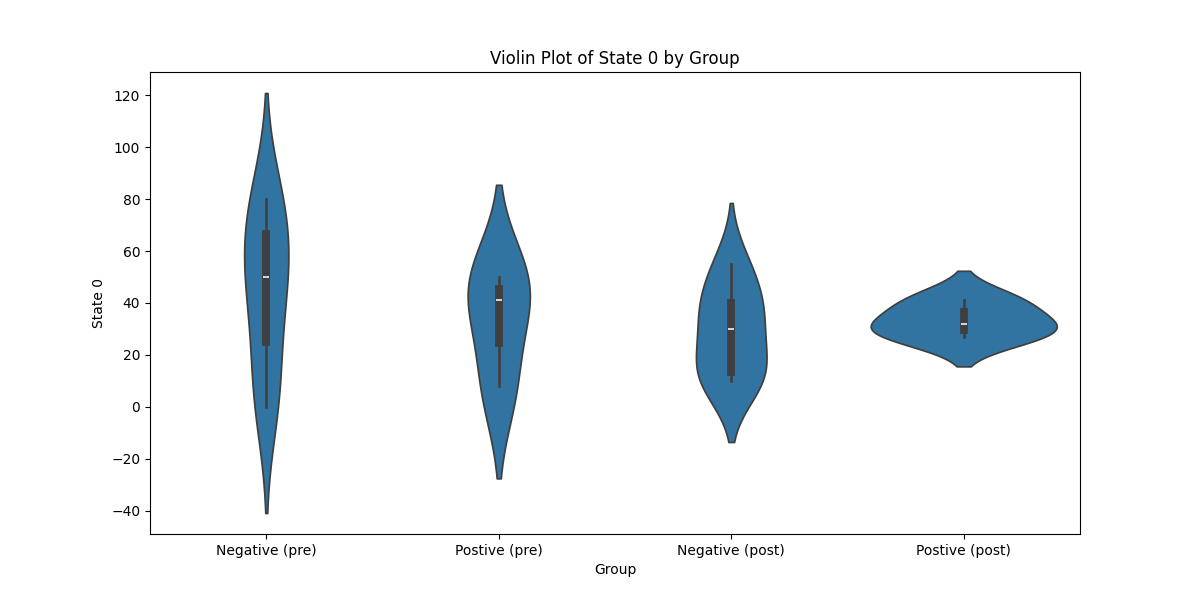


Figure S2.2-1 Differences in State 0 between pre-tDCS and post-tDCS.

Positive: responders; Negative: non-responders


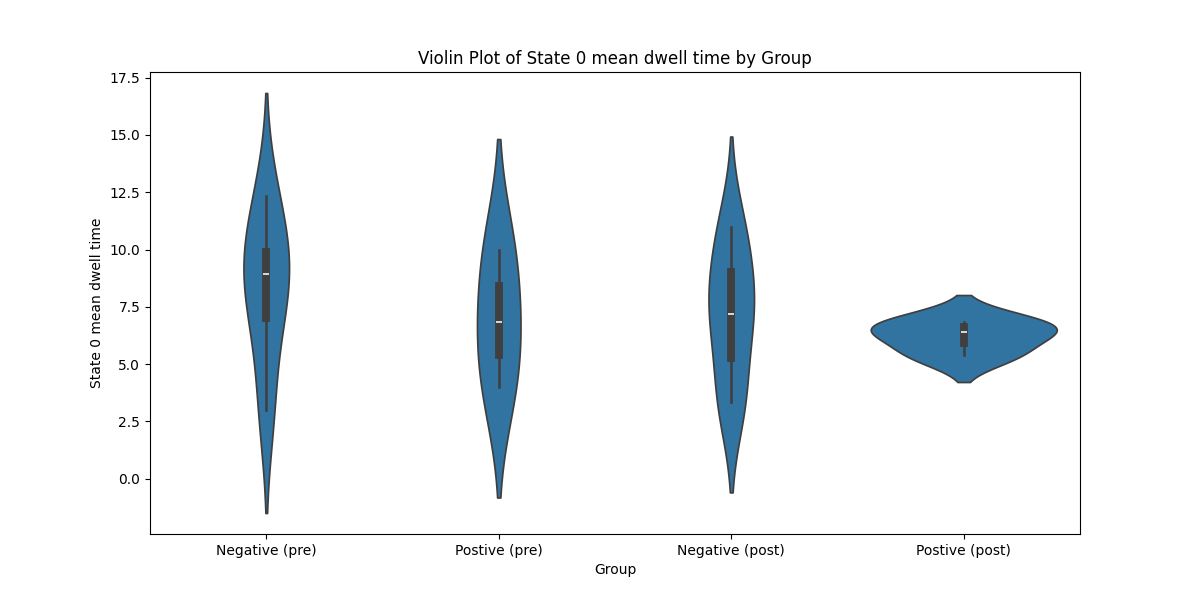


Figure S2.2-2 Differences in State 0 dwell time between pre-tDCS and post-tDCS.

Positive: responders; Negative: non-responders


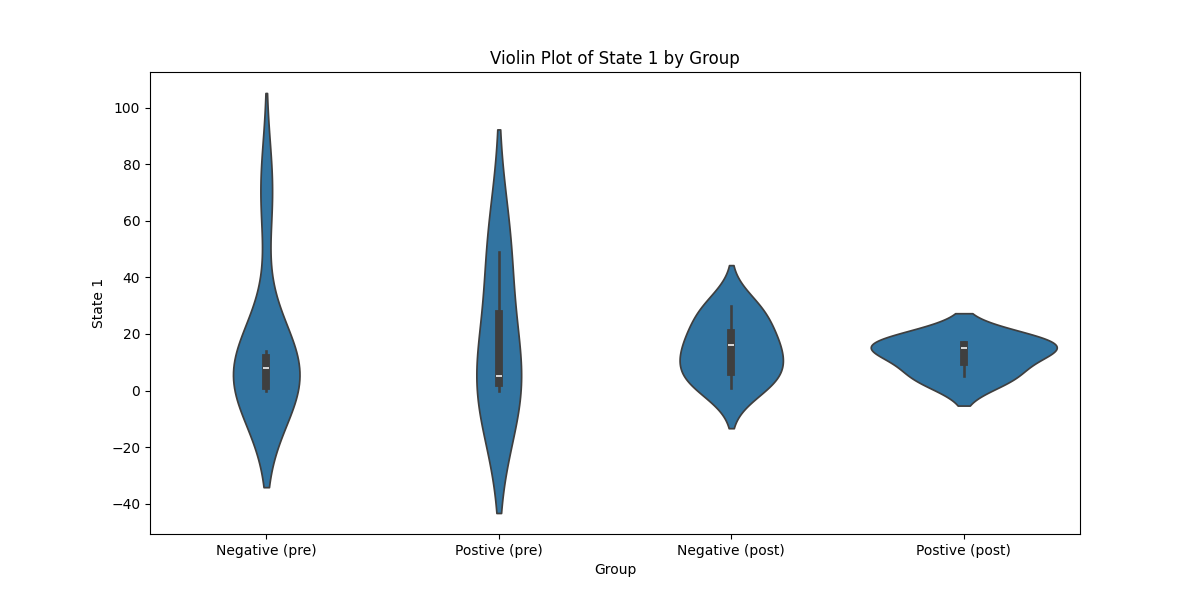


Figure S2.2-3 Differences in State 1 between pre-tDCS and post-tDCS.

Positive: responders; Negative: non-responders


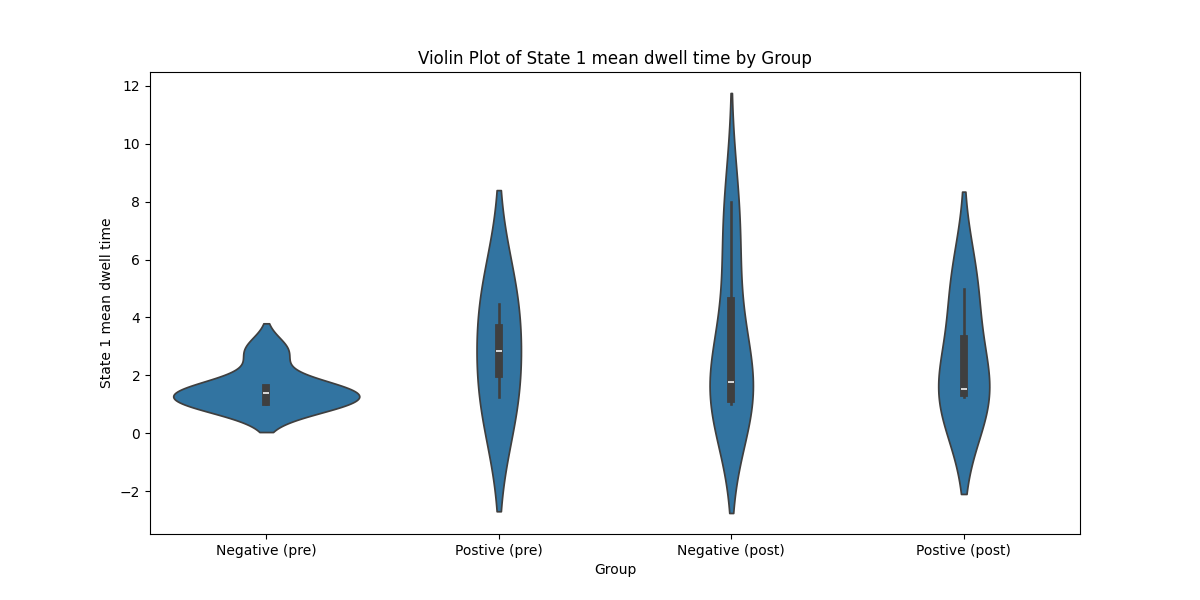


Figure S2.2-4 Differences in State 1 dwell time between pre-tDCS and post-tDCS.

Positive: responders; Negative: non-responders


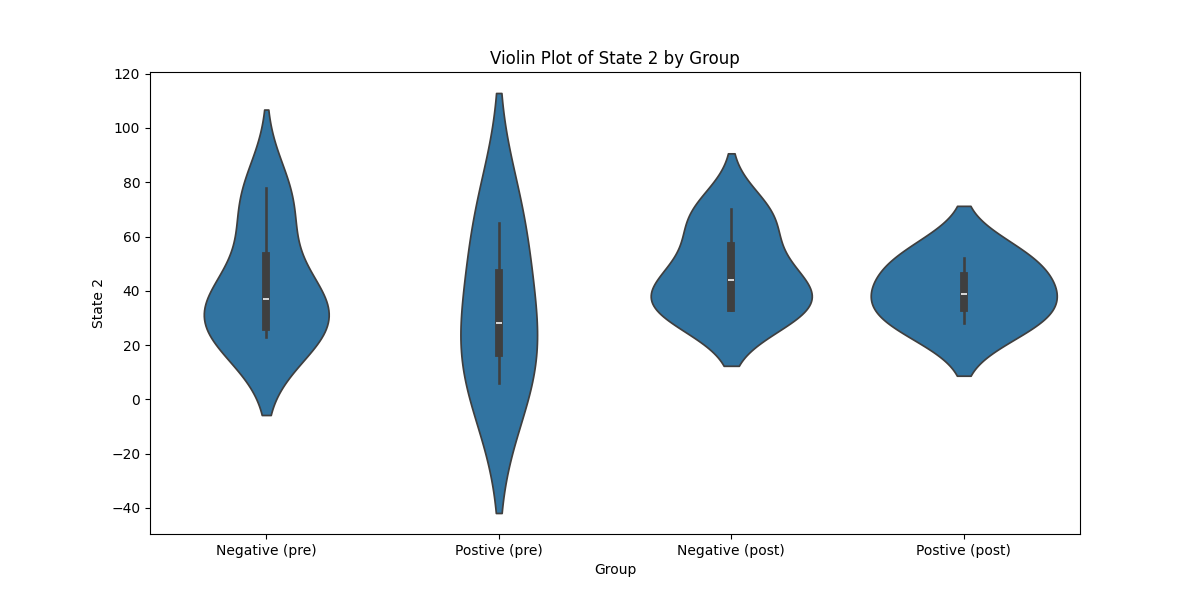


Figure S2.2-5 Differences in State 2 between pre-tDCS and post-tDCS.

Positive: responders; Negative: non-responders


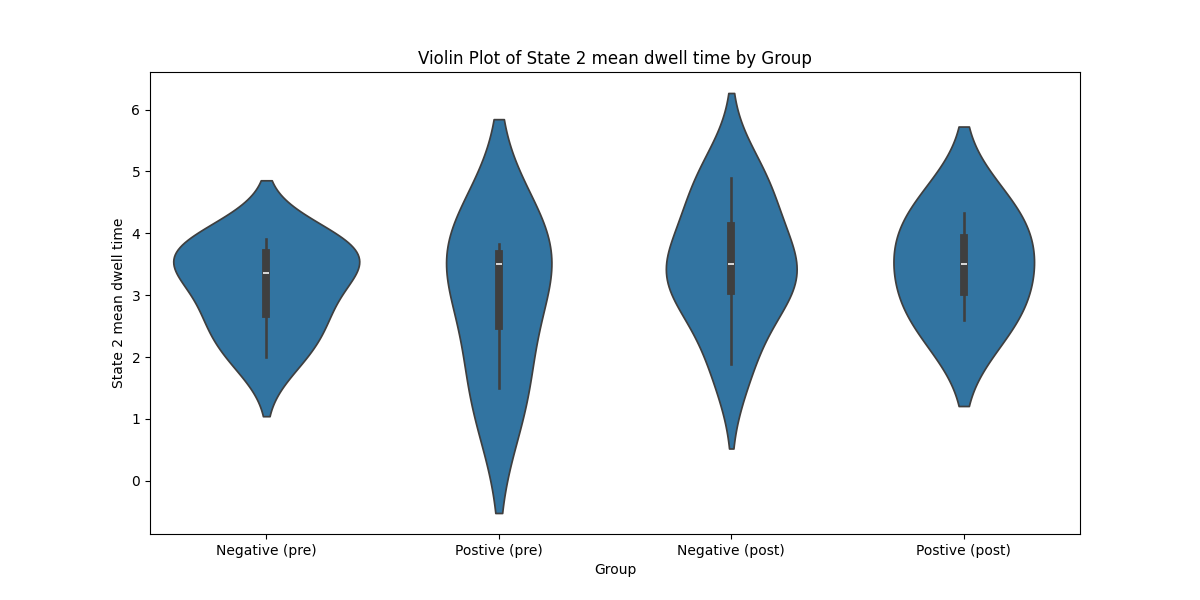


Figure S2.2-6 Differences in State 2 dwell time between pre-tDCS and post-tDCS.

Positive: responders; Negative: non-responders


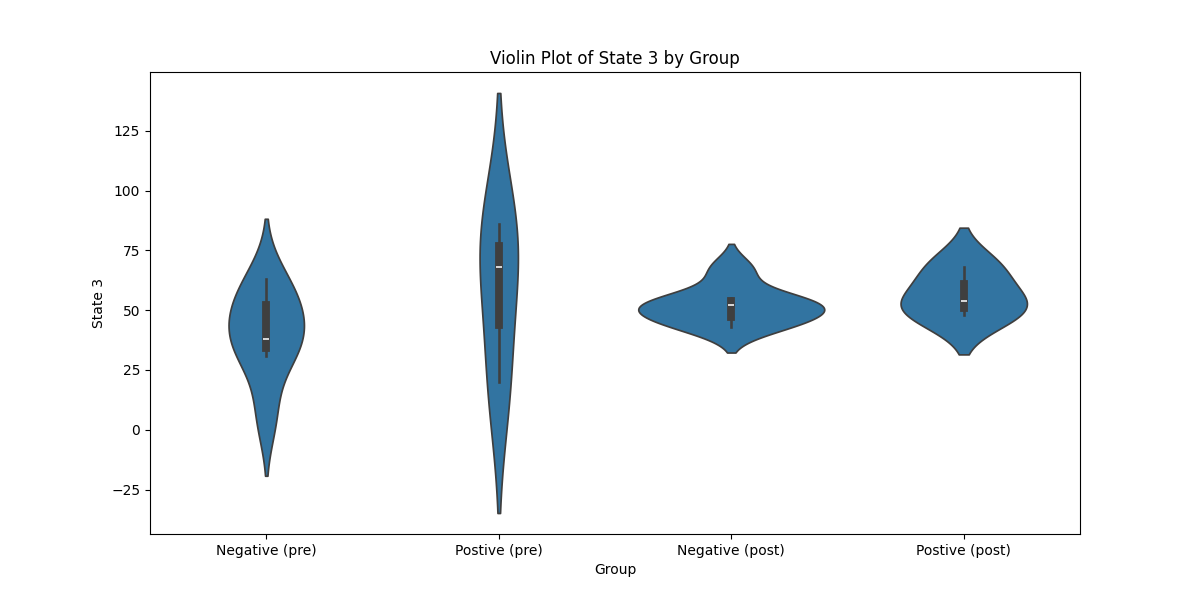


Figure S2.2-7 Differences in State 3 between pre-tDCS and post-tDCS.

Positive: responders; Negative: non-responders


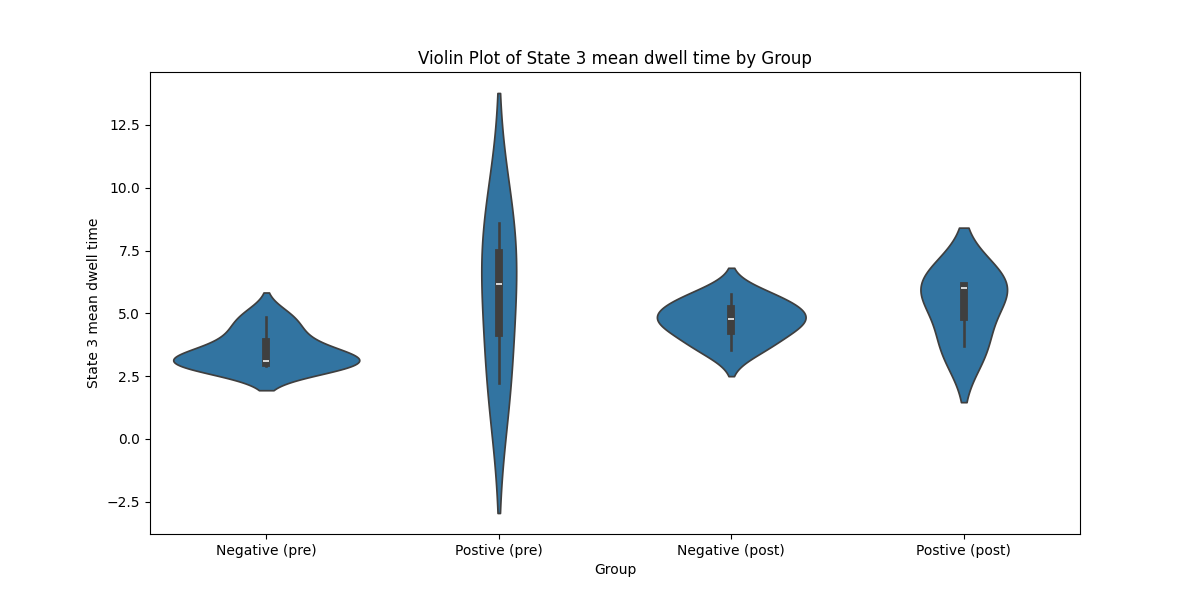


Figure S2.2-8 Differences in State 3 dwell time between pre-tDCS and post-tDCS.

Positive: responders; Negative: non-responders


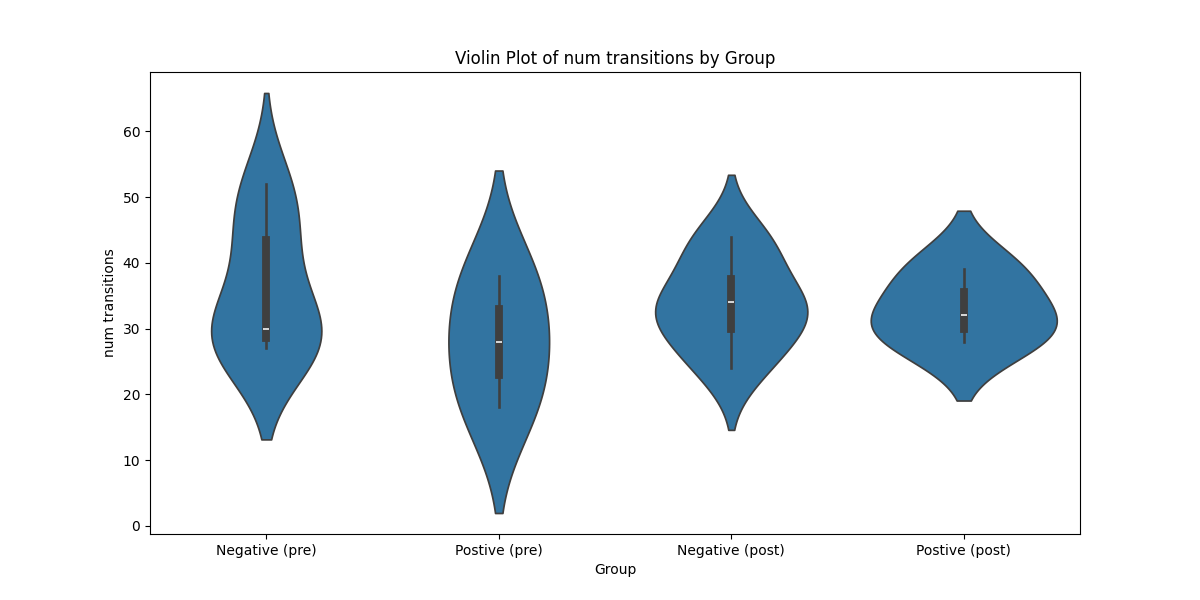


Figure S2.2-9 Differences in transition number between pre-tDCS and post-tDCS.

Positive: responders; Negative: non-responders

- 1. Pre-tDCS vs HC


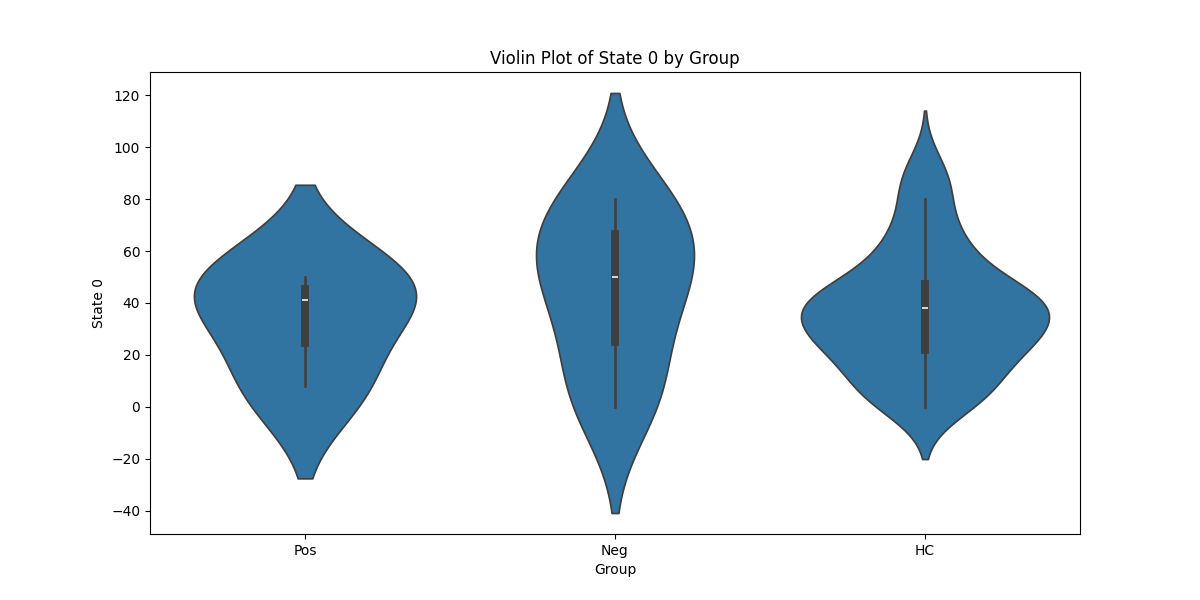


Figure S2.3-1 Differences in State 0 among responders, non-responders, and HCs in pre-tDCS data.

Pos: responders, Neg: non-responders, HC: healthy controls.


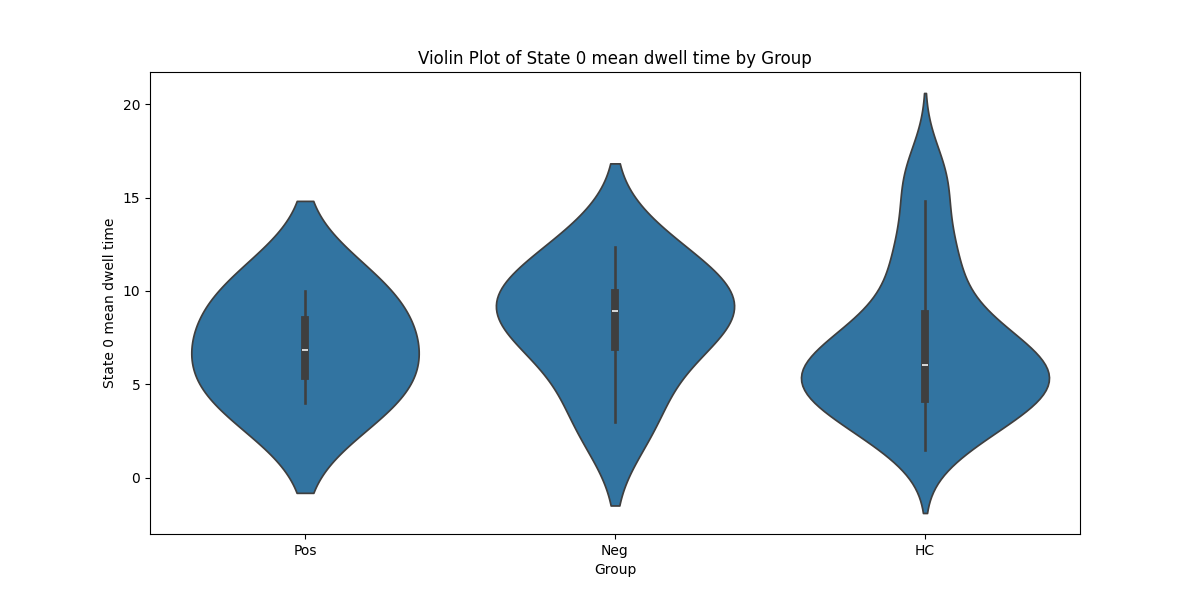


Figure S2.3-2 Differences in State 0 dwell time among responders, non-responders, and HCs in pre-tDCS data.

Pos: responders, Neg: non-responders, HC: healthy controls.


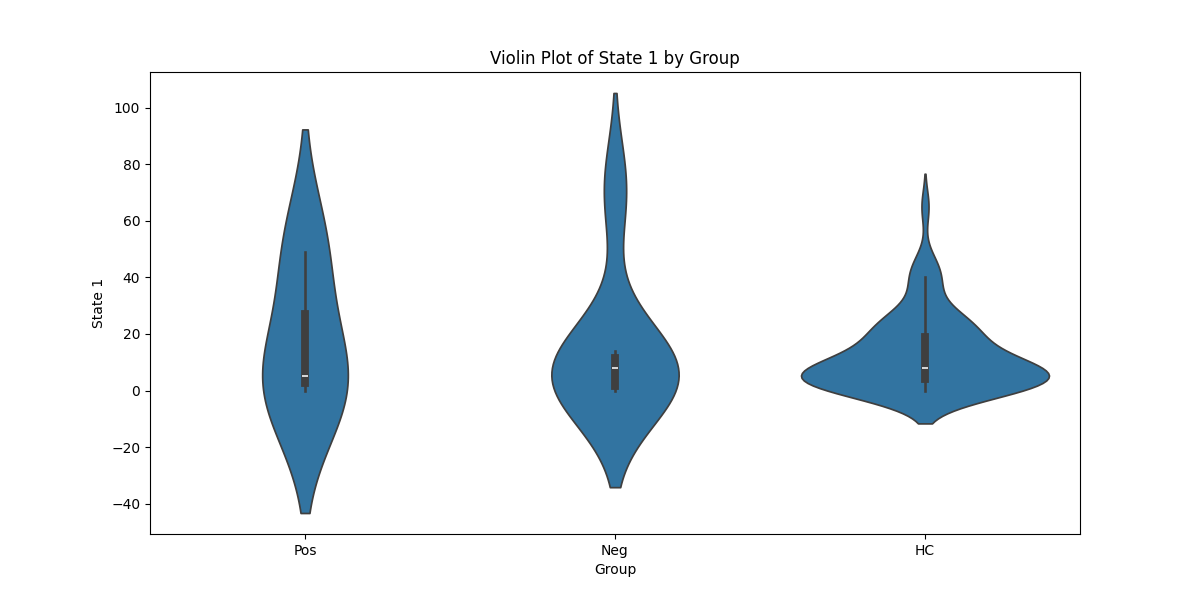


Figure S2.3-3 Differences in State 1 among responders, non-responders, and HCs in pre-tDCS data.

Pos: responders, Neg: non-responders, HC: healthy controls.


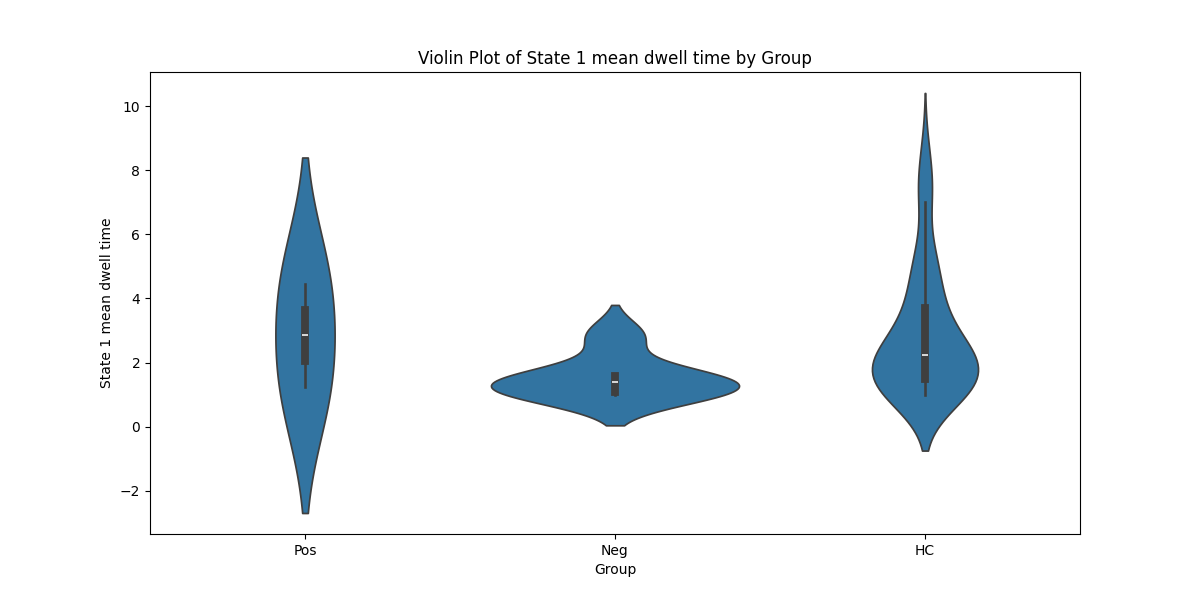


Figure S2.3-4 Differences in State 1 dwell time among responders, non-responders, and HCs in pre-tDCS data.

Pos: responders, Neg: non-responders, HC: healthy controls.


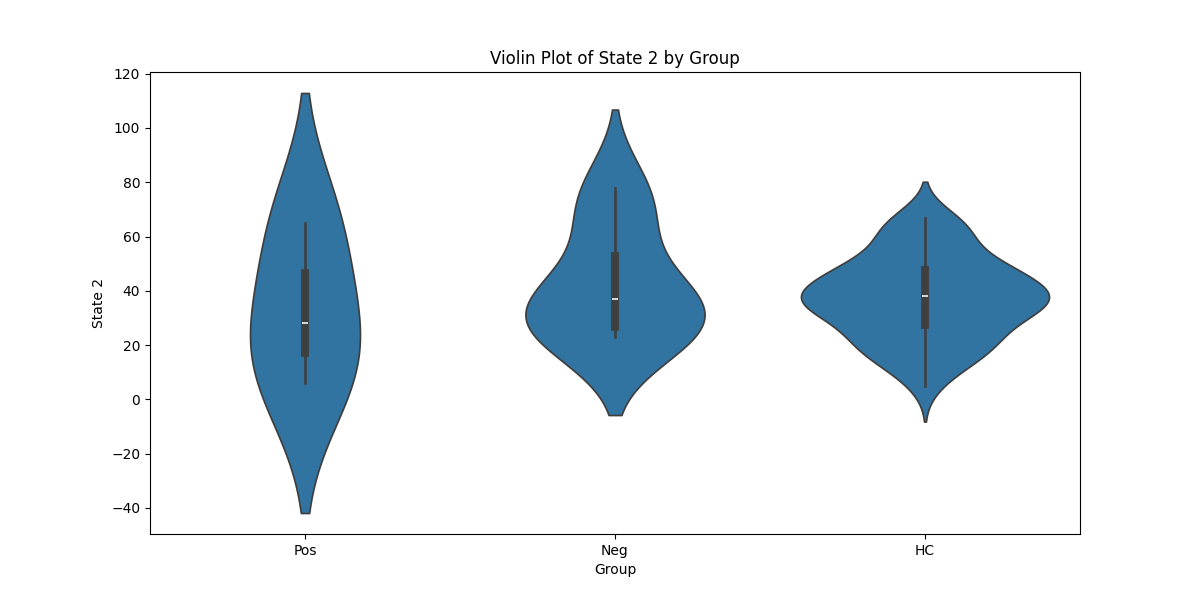


Figure S2.3-5 Differences in State 2 among responders, non-responders, and HCs in pre-tDCS data.

Pos: responders, Neg: non-responders, HC: healthy controls.


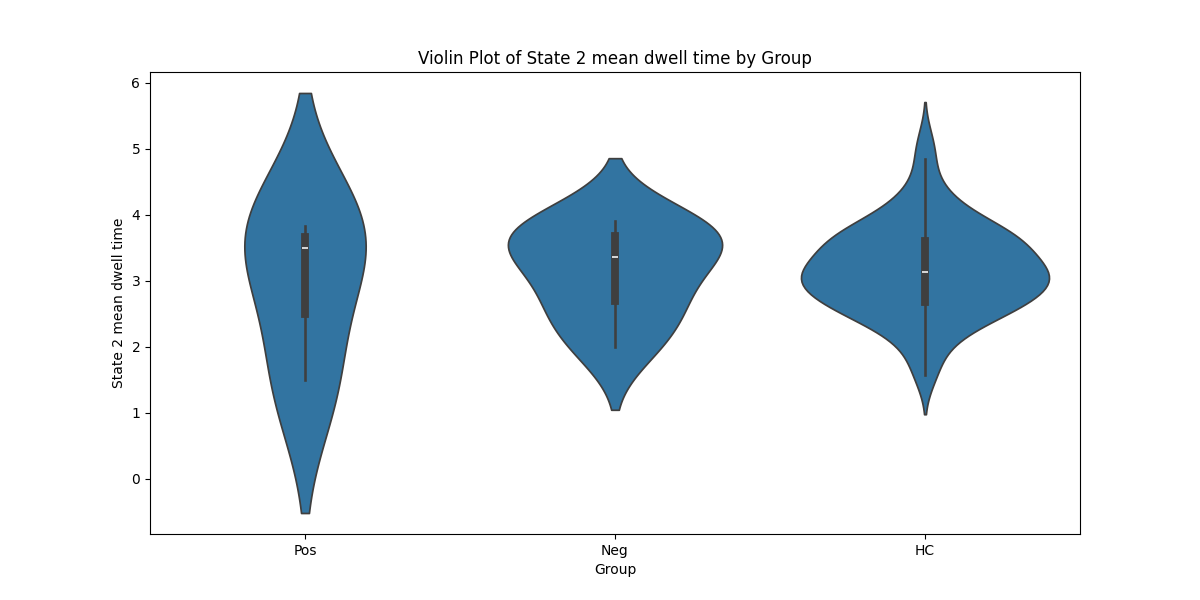


Figure S2.3-6 Differences in State 2 dwell time among responders, non-responders, and HCs in pre-tDCS data.

Pos: responders, Neg: non-responders, HC: healthy controls.


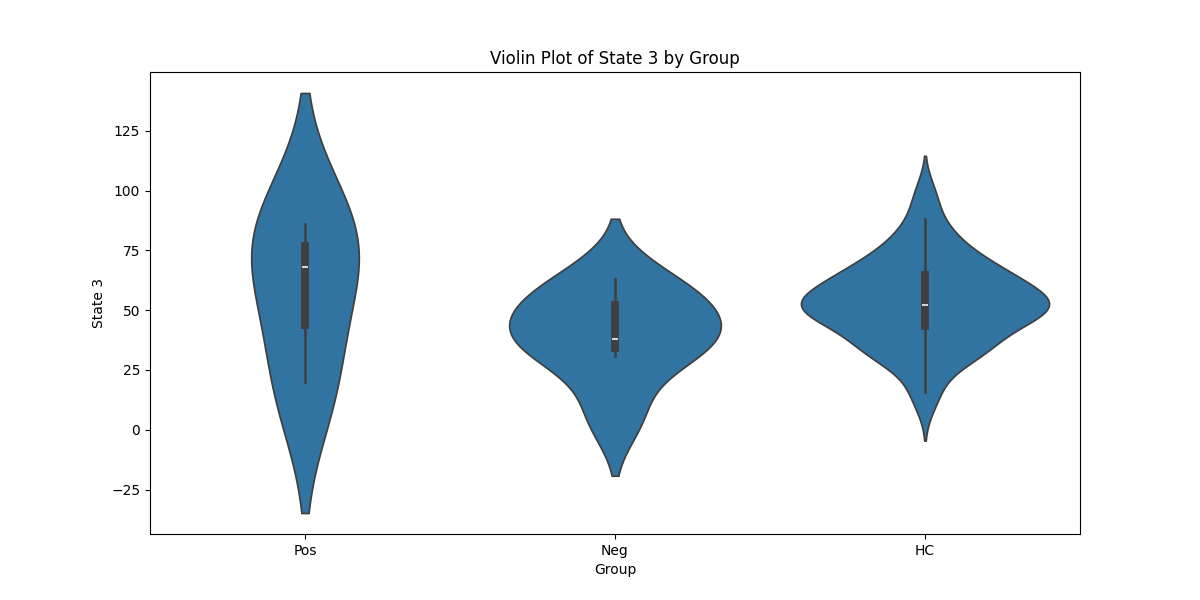


Figure S2.3-7 Differences in State 3 among responders, non-responders, and HCs in pre-tDCS data.

Pos: responders, Neg: non-responders, HC: healthy controls.


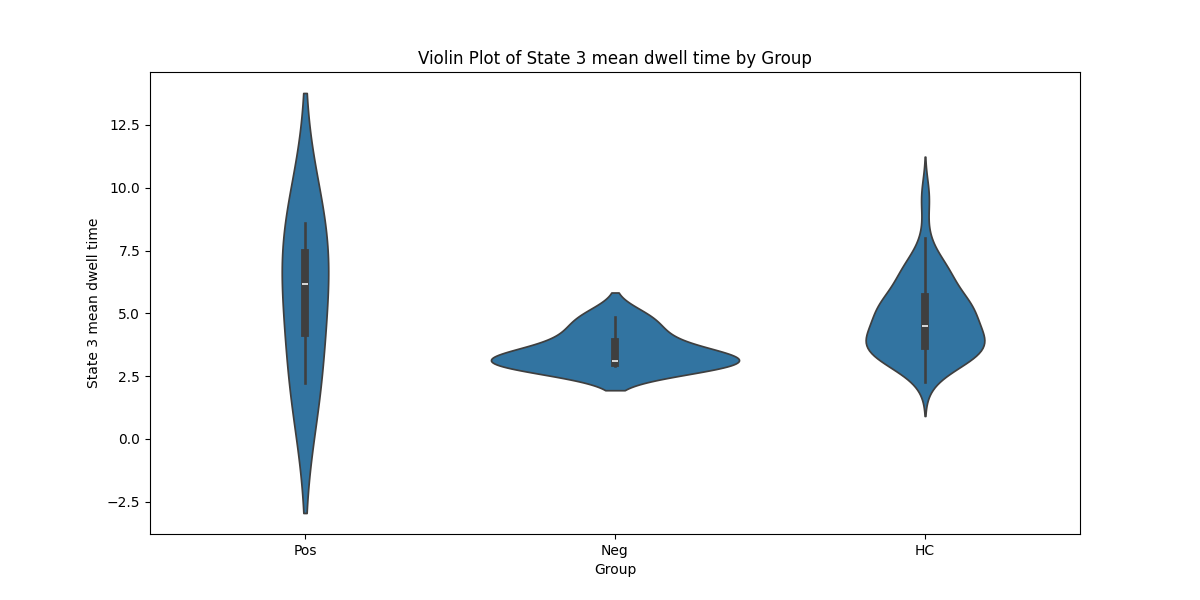


Figure S2.3-8 Differences in State 3 dwell time among responders, non-responders, and HCs in pre-tDCS data.

Pos: responders, Neg: non-responders, HC: healthy controls.


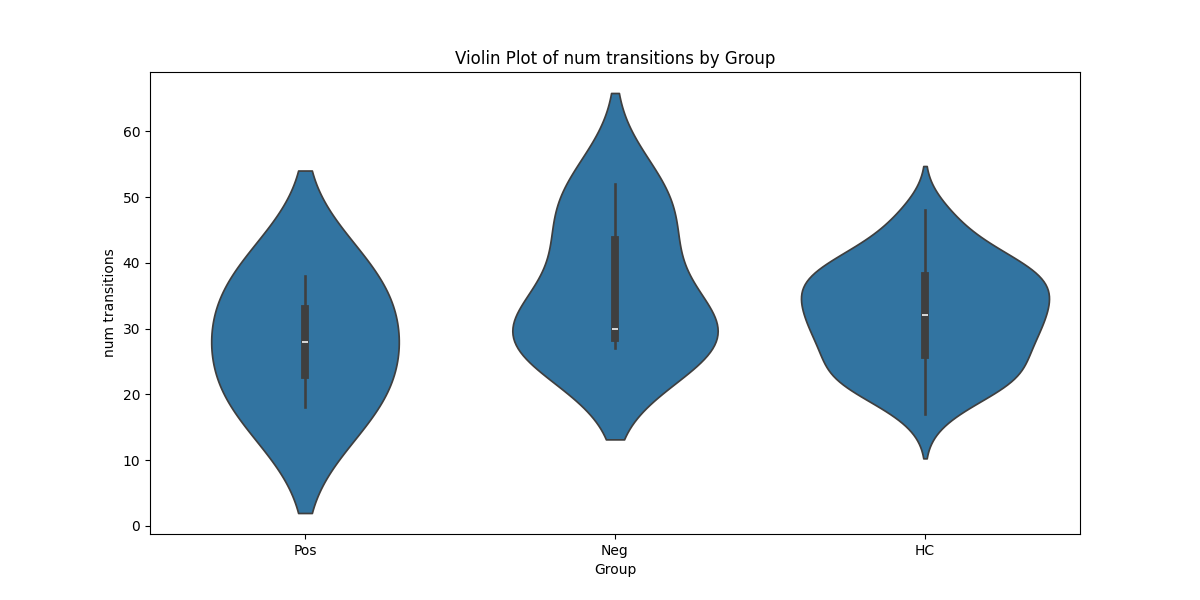


Figure S2.3-9 Differences in transition number among responders, non-responders, and HCs in pre-tDCS data.

Pos: responders, Neg: non-responders, HC: healthy controls.
